# Transcription factor repertoire in Ashwagandha (*Withania somnifera*) through analytics of transcriptomic resources: Insights into regulation of development and withanolide metabolism

**DOI:** 10.1038/s41598-017-14657-6

**Published:** 2017-11-30

**Authors:** Sandhya Tripathi, Rajender Singh Sangwan, Lokesh Kumar Narnoliya, Yashdeep Srivastava, Bhawana Mishra, Neelam Singh Sangwan

**Affiliations:** 1 Department of Metabolic and Structural Biology, CSIR-Central Institute of Medicinal and Aromatic Plants (CSIR-CIMAP), Lucknow, 226015 India; 2 Center of Innovative and Applied Bioprocessing (A National Institute under Department of Biotechnology, Govt. of India), Sector-81 (Knowledge City), PO Manauli, S.A.S. Nagar, Mohali, 140306, Punjab India; 3 Academy of Scientific and Innovative Research (AcSIR) (An Institution of National Importance by an Act of Parliament),, AcSIR Campus, CSIR-HRDC, Sector-19, Kamla Nehru Nagar, Ghaziabad, Ghaziabad, 201002 Uttar Pradesh India

## Abstract

Transcription factors (TFs) are important regulators of cellular and metabolic functions including secondary metabolism. Deep and intensive RNA-seq analysis of *Withania somnifera* using transcriptomic databases provided 3532 annotated transcripts of transcription factors in leaf and root tissues, belonging to 90 different families with major abundance for WD-repeat (174 and 165 transcripts) and WRKY (93 and 80 transcripts) in root and leaf tissues respectively, followed by that of MYB, BHLH and AP2-ERF. Their detailed comparative analysis with *Arabidopsis thaliana*, *Capsicum annum*, *Nicotiana tabacum* and *Solanum lycopersicum* counterparts together gave interesting patterns. However, no homologs for WsWDR representatives, LWD1 and WUSCHEL, were observed in other Solanaceae species. The data extracted from the sequence read archives (SRA) in public domain databases were subjected to re-annotation, re-mining, re-analysis and validation for dominant occurrence of *WRKY* and *WD-repeat* (*WDR*) gene families. Expression of recombinant LWD1 and WUSCHEL proteins in homologous system led to enhancements in withanolide content indicating their regulatory role *in planta* in the biosynthesis. Contrasting expression profiles of WsLWD1 and WsWUSCHEL provided tissue-specific insights for their participation in the regulation of developmental processes. The in-depth analysis provided first full-spectrum and comparative characteristics of TF-transcripts across plant species, in the perspective of integrated tissue-specific regulation of metabolic processes including specialized metabolism.

## Introduction

Plants are sessile in nature and distinct to animals as they have to respond to stresses in a different manner. When they are exposed to multifarious types of unfavorable environmental conditions such as drought, wounding, salinity, cold and nutrient starvation etc., they develop circuitous mechanisms at cellular and molecular levels to counter these adverse conditions enabling their adaptation to such conditions^1,2^. The manifestations in elemental biological processes and appropriate development require constitutive expression of some of the genes on one hand while spatio-temporal pattern specific expression of other genes. This is basically guided by the relevant communication of transcription factors (TFs) either with some other TFs or with *cis*-acting elements resulting in different cellular responses. The totality and multiplicity of TFs is directly correlated with complexity of an organism by being involved in regulation of gene expression^1^. Based on the combinatory control of protein-protein interactions, TFs may consecutively act as activators of a set of genes on one hand or as repressors on the other^2,3^. The basic TF organization consists of a DNA-binding domain (DBD) involved in interaction with the *cis*-regulatory elements of the target genes in addition to the domain for protein- protein interaction which is responsible for oligomeric interactions between these modular proteins (TFs) and other regulators^3,4^. According to the previous studies, ~7% of coding portion of plant genome sequences function as transcription factors^5,6^. Therefore, full-spectrum mining and in depth studies of transcription factors and their regulation will advance our knowledge on basic biological processes in the tests species. In addition, such knowledge is a pre-requisite for the development of metabolically engineered varieties.

Recently, efficient and high-throughput sequencing technologies and platforms have led to almost exponential accumulation of genomic and transcriptomic sequences in public domain databases^7–12^. It has emerged as one of the powerful resources to examine a variety of sets of genes of a particular species of interest. Evaluation of quantitative expression of transcribed genes specific to an organ/tissue facilitated through RNA-seq is, thus, considered to be a robust tool for determination of differentially expressed transcripts under different conditions and for different processes^13^.

Current status of information regarding TFs in *W. somnifera* is almost limited to *WRKY* genes for which transcriptional regulation of pathway genes has been observed due to higher levels of corresponding gene transcripts^14^. Although, biosynthetic pathways for the major secondary metabolites of *W. somnifera* have previously been characterized,^9–11,15–18^ the insights into their underlying regulatory mechanisms remain chiefly unexplored. The involvement of MVA and DOXP pathways for withanolide biosynthesis and other key factors have also been proposed in our earlier studies^18–21^. Though, through earlier reports, some aspects of transcription factors (TF) were elucidated in general^9,10^ and substantial transcriptomic sequences resources have become available on the plant., nevertheless, considering the scale of their occurrence, major task of generating nearly full spectrum TF dataset remains due for the species. Accordingly, there are substantial gaps in terms of knowledge of their regulatory roles and mechanisms for the plant.

The major potential pharmacological characteristics of the plant are aphrodisiac, anti-tumerogenic, rejuvenating, anti-inflammatory, anti-stress, anti-cancer, anti-diabetic, antioxidant, neuroprotective and immunomodulation^22–26^. Although, the plant possesses alkaloids and glycosides, it has the most characteristic feature of synthesizing diverse and abundant levels of specialized phytochemicals called withanolides which are basically triterpenes ancestry steroidal lactones built on ergostane skeleton^27–30^. Various pharmacological applications of the medicinal plant have been attributed to withanolides of the herb. Therefore, investigation of genes and other aspects of withanolide biosynthetic pathway have been our first attention of investigation with eventual aim to achieve enhanced metabolites (withanolides) content *in planta* ^20,21,31–34^. Though, with our work on withanolide biosynthetic pathway,^20,21,34^ Ashwagandha has become the most well investigated Indian medicinal plant with respect to its withanolide pathway related biochemistry and molecular biology, however literature on the plant is limited ^14^ with respect to TF medicated regulation of its metabolic pathways for the biosynthesis of its specialized phytochemicals including withanolides. There are a number of transcription factors for example- WRKY, WDR, MYB, BHLH, AP2/ERF and each of the TF can influence/regulate entire or subset(s) of the pathway/process^3–6,35^.

Therefore, this study is aimed at gene mining from leaf and root transcriptomic sequence resources available for *Withania somnifera* to divulge full-spectrum of TFs through powerful analytics as an approach combined with detailed gene analysis *in planta* and validate/understand regulation of biosynthetic routes and developmental processes in the plant. This is the first detailed account of TF repertoire from *W. somnifera* as well as first report on elucidation of *WDR* TF gene family namely *WsLWD1* and *WsWUSCHEL* along with functional analysis. The study will facilitate the understanding and regulation of withanolide production as specialized metabolism in *Withania* *somnifera* *vis-à-vis* regulatory mechanism of gene expression in other plant species of medicinal significance.

## Material and Methods

### Retrieval and processing of leaf and root transcriptome datasets

Transcriptome data used in the study were obtained from NCBI data resource (https://www.ncbi.nlm.nih.gov/) available as SRA053485 and transcripts of significant lengths were obtained for different transcription factors after appropriate processing of the raw sequence files and their assembly using different bioinformatics tools.

### Determination of transcription factors, GO assignment and comparison with other species

The assembled transcripts for the two tissues were subjected to BlastX analysis at non-redundant database, GO annotation, enzyme code classification and assignment to KEGG pathways using Blast2GO 2.8 available online at default parameters^36^. Homology search was performed against *A. thaliana* TF database (http://arabidopsis.med.ohio-state.edu/AtTFDB/) and Plant TF database (Plant TFDB) (http://planttfdb.cbi.pku.edu.cn/) for different members of Solanaceae at default parameters.

### Phylogenetic analysis

The nucleotide sequences for *V. vinifera* transcription factors namely WRKY and WDR were downloaded from TreeTFDB (http://treetfdb.bmep.riken.jp/download.pl) and NCBI at Genebank (http://www.ncbi.nlm.nih.gov/gene). The clustering of transcripts in the tree was done using MEGA 6.06^37^ by means of maximum likelihood method^38^.

### Expression analysis of *WDR* (*WsLWD*1 and *WsWUSCHEL*) TFs

Primers specific to gene amplicon were designed for *WsLWD1* and *WsWUSCHEL* transcription factors to perform quantitative real time PCR (qRT-PCR) analysis. Candidate gene expression was normalized against β-actin. Total cDNA was synthesized from 5 µg of RNA (DNase treated) using different tissues like berry, flower, leaf, root and stem. Both quantitative and semi-quantitative PCR analysis was performed for these genes as done previously^8,18^. Semi-quantitative PCR reaction was performed in volume of 20 µl using template (first strand cDNA), Taq DNA polymerase (0.5 U) and each primer (10 picomoles) using PCR cycle as follows: 94 ^°^C (3 min), 94 ^°^C (30 s) 30 cycles, 50 ^°^C (40 s) and 72 ^°^C (2 min) followed by a 72 ^°^C (7 min) as final extension in a thermal cycler (Eppendorf). For quantitative assay, reaction mix was made to 20 µl of total reaction volume, consisting of ~100 ng template (cDNA), 10 µl (SYBR Green) ROX master mix (ABI Biosystems, USA) and 5 pM (gene specific primer each). Triplicate sets of reactions were carried out at default conditions as done earlier^8,18^. Gene expression normalization was done against β-actin (endogenous control) and expression levels (relative) indicated as relative quantification (RQ) was calculated by applying ΔΔC_T_ method.

### Isolation and cloning of *WDR* (*WsLWD1* and *WsWUSCHEL*) TFs

To isolate full length cDNA of *WDR* *(WsLWD1*and *WsWUSCHEL)*, primers were designed using sequences identified from transcriptome to amplify amplicons of 1150 bp and 786 bp corresponding to *WsLWD1* and *WsWUSCHEL* respectively. PCR was accomplished in a reaction volume of 50 µl using 1.0 µl template (cDNA), 2 µl of primers each 10 picomole and master mix 45 µl. Fragments on amplification were analyzed through 1% agarose gel, desired size amplicons were sliced, eluted and cloned in pJET1.2 cloning vector. Colonies which got transformed were identified *via* colony PCR and subjected to plasmid isolation. Full length *WDR* TFs (*WsLWD1 *and *WsWUSCHEL*) were further confirmed by restriction digestion, sequencing and homology matching with sequences from transcriptomic data.

### Sequence and phylogenetic analysis of resulting proteins

NCBI BlastX tool (http://blast.ncbi.nlm.nih.gov/Blast/) was used for searching similarities between WDR TFs (*WsLWD1 *and *WsWUSCHEL*) and similar transcription factor sequences from other plants in the public database. Evolutionary relationships of *WsLWD1* and *WsWUSCHEL* transcription factors were assessed with similar sequences from other plants *via* constructing a phylogenetic tree using MEGA 6.06 software (http://www.megasoftware.net) through application of maximum likelihood method.

### Cloning of WDR (*WsLWD1* and *WsWUSCHEL*) TFs in plant expression vector and *in planta* transformation

Primers were designed from full length *WsLWD1* and *WsWUSCHEL* sequences and full length cDNA containing restrictions sites were subsequently amplified. A high efficacy Pfu DNA polymerase from Fermentas to amplify full length cDNAs for *WsLWD1* and *WsWUSCHEL* with PCR conditions as; 94 ^°^C (3 min), 94 ^°^C (40 s) 35 cycles, 55 ^°^C (1 min) and 72 ^°^C (3 min) with final extension 72 ^°^C (7 min) afterwards in a thermal cycler PCR. The amplified products were subjected to cloning in pJET1.2 vector followed by transformation in *E. coli* DH5α host cells. Colonies containing positive clones using colony PCR were imposed for plasmid isolation along with pBI121 vector. Constructs *WsLWD1*-pBI121 and *WsWUSCHEL*-pBI121 were made by ligation of appropriate digested products using T4 ligase enzyme. These constructs were transformed in *E. coli* cells and plasmids were isolated containing *WsLWD1*-pBI121 and *WsWUSCHEL*-pBI121 constructs. Positive clones were further used for transformation in *A. tumefaciens*.

### Transient transformation and analysis of *WsLWD1*-pBI121 and *WsWUSCHEL*-pBI121 constructs in *W. somnifera*

Transient transformation being an economical and fast method to ratify the impact of gene expression was used to transiently overexpress the two transcription factors encoding genes namely *WsLWD1* and *WsWUSCHEL* in *W.somnifera*. The transformation was performed as per our earlier report^39^. The PCR conditions used were: 94 °C (3 min), 94 °C (30 s), 55–58 °C (30 s), 35 cycles of 72 °C (1 min), with final extension at 72 °C (7 min). Actin was considered as an internal control, and expressions of both genes were visualized on 1% agarose gel (Table S1). Further, to validate semi-quantitative PCR results, real time quantitative PCR was accomplished to quantify the transcripts of *WDR* (*WsLWD1* and *WsWUSCHEL*) in transformed tissues. For this, same cDNA of semi-quantitative PCR analysis was used. Real time PCR was performed for transiently transformed tissues using SYBR green as florescent dye. The reactions were performed in triplicates (10 µl volume), using gene specific primers for both the transcription factors along with actin gene as an endogenous control for each sample. The other conditions were kept as mentioned above in real time PCR section.

### HPLC analysis of withanolide content in transiently transformed *W. somnifera*

Withanolides were extracted and estimated according to methods developed in our lab. The isolated enriched fractions were analysed for withanolides by HPLC as earlier^40^. Briefly, HPLC system (Waters, USA) attached with quaternary pump, pump controller, auto-sampler, photodiode array detector (Model 996), was used along with reverse-phase (RP). Withanolides were eluted by binary gradient using mobile phase of HPLC grade water and solvent in a gradient manner. Each chromatogram was generated and the whole analysis was subjected to computations by Empower software embedded in Waters HPLC for parametric validation as performed earlier^40^.

## Results

### Mining of transcripts coding for transcription factor genes in *W. somnifera*

**Figure 1:**
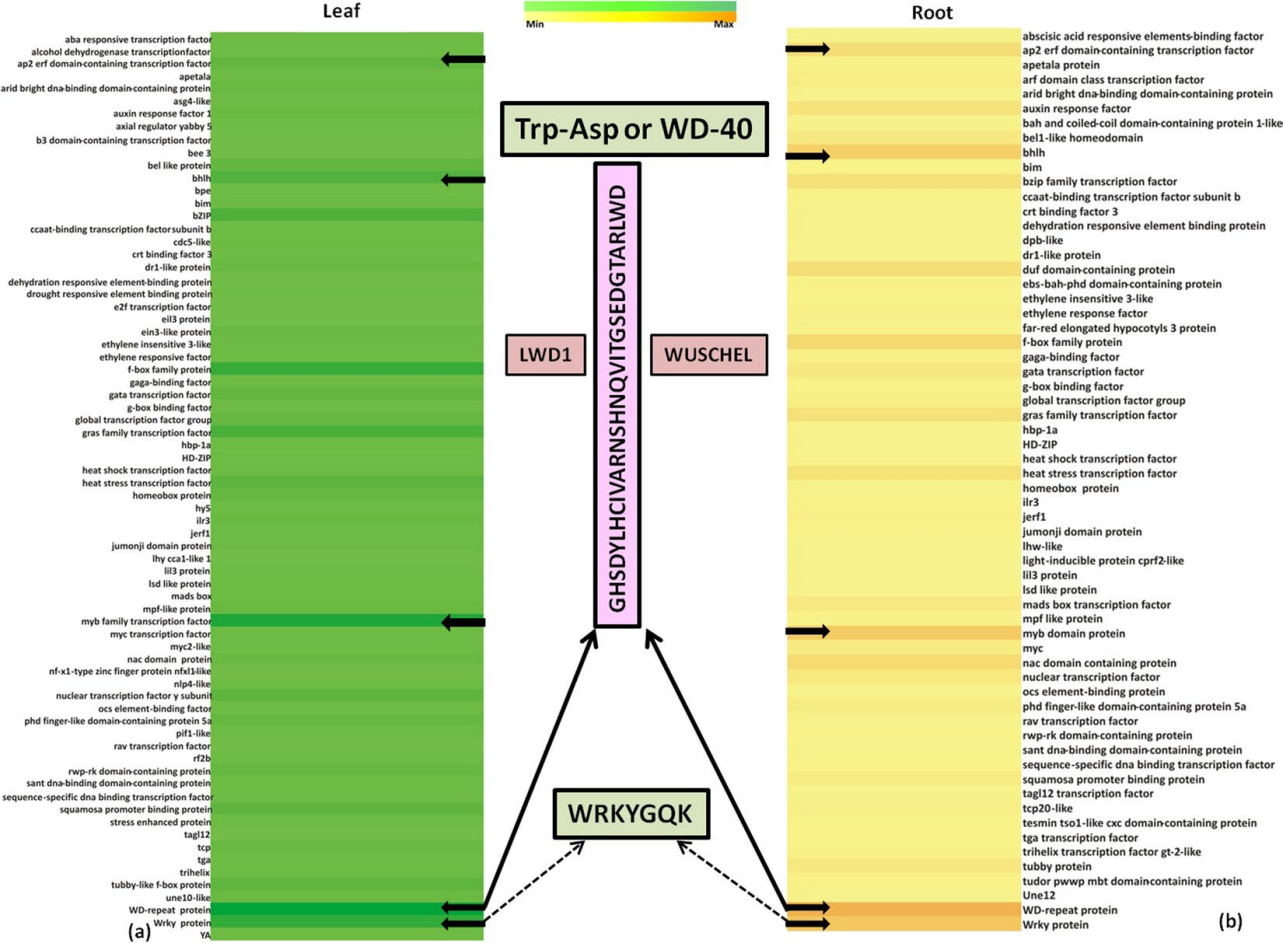
Distribution of the TF families in *W.somnifera* tissues. The abundance of transcripts for different TF families in *W.somnifera* **(a)** leaf and **(b)** root tissues is represented. The color bar provides the scale for the expression pattern from minimum to maximum based on the number of transcripts assigned to a particular gene family. The TFs namely- WRKY and WDR protein which are studied in detail in the current study have been highlighted with their conserved domains and the two representative members of WDR proteins namely, LWD1 and WUSCHEL which have been undertook in the study are shown. The characteristic motif for the two genes has been shown in blocks. Additionally other TFs having significant abundance are shown with black arrows.

In the previous report^9^, the leaf and root transcriptomes of NMITLI-101 were analyzed and the glimpses of overall transcription factor repertoire present in the transcriptome data were provided in addition to the transcript related to overall metabolic processes. In the current study, we have made an intensive effort to sample nearly complete-spectrum of sets of different TFs of *W. somnifera* and functionally categorize some of them in detail in the perspective of different biological processes including withanolide-specialized metabolic processes using earlier sequence data resource available in public domain majorly as sequence read archives (SRAs)^9,10^. A total of 834080 leaf ESTs and 721780 root ESTs with average read length of approximately 312 bp and 283 bp respectively were obtained through SRA database. After processing, 120458 high quality reads were obtained which were further preceded for downstream analysis into contigs and singletons containing some overlap fragments. These sequence analysis results were annotated using B2G to collect the major annotations for transcription factors (Fig. 1).

Together 3532 putative TF encoding transcripts assigned to about 90 families, were identified, which represented approximately 3% of the total *W. somnifera* transcriptomic dataset. The transcripts comprising GO terms related to transcriptional regulation were retrieved and subjected to analyze expression of different gene families differentially in the two tissues (Table S2). Based on the observation of the distribution of transcripts for different transcription factors, maximum numbers of transcripts were found for WDR transcription factors (165 leaf and 174 root tissues, respectively). Although, significant presence of WRKY, BHLH, F-BOX, BZIP, AP2 and AP2-ERF domain protein were also found commonly in both the tissues. Noteworthy, presence of transcripts for ethylene response factor, GATA, GRAS, HOMEOBOX protein, heat shock/stress transcription factor, MYB and NAC etc. were also observed. NAC, MADS box and DUF proteins were however shown to be more significantly expressed in root tissues as opposed to leaf tissues. Contrarily, squamosa promoter binding protein was likely to be more significantly expressed in leaf tissues (Fig. 1).

### Comparative profiling of transcription factors

**Figure 2:**
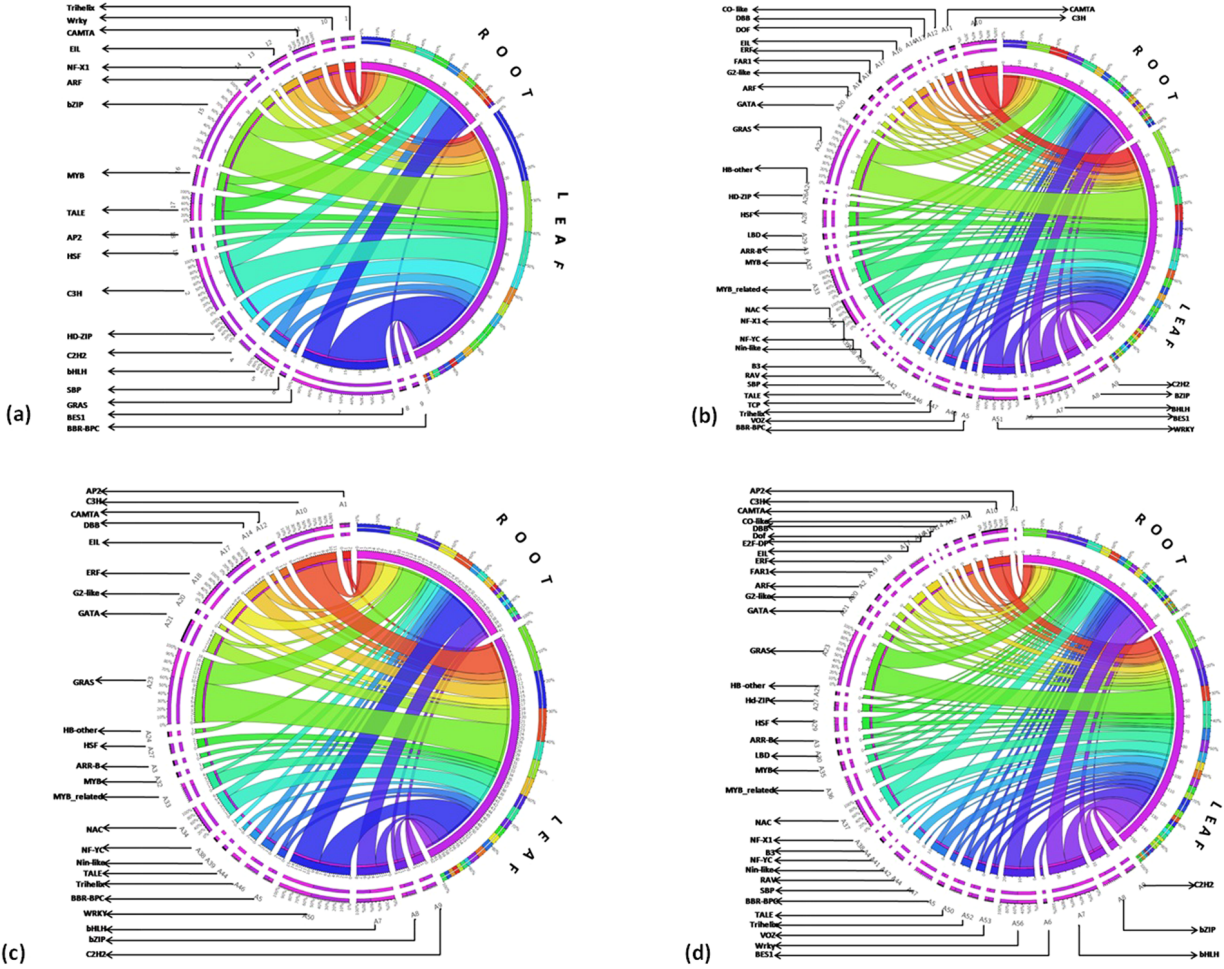
Comparison of *W.somnifera* TF dataset with different species. **(a)** Transcripts from leaf and root dataset were compared with TFs of model plant *A. thaliana* initially. Further, transcript comparison of *W.somnifera* transcription factors with three Solanaceae plant species transcription factors **(b)** *C. annum*, **(c)** *N. tabacum* and **(d)** *S.lycopersicum* is shown.

To understand the biological roles of a gene in a plant species *via-a-vis* members across the plant kingdom, gathering of information on amount and related diversity is an important domain of study. The whole transcriptome data sets for both leaf and root tissues was re-annotated and re-analyzed against *A.thaliana*, *S. lycopersicum*, *N. tabacum* and *C. annum* transcription factor data obtained from Plant TFDB^41^ to gain insights into species specific transcription factors by local BlastX program at default parameters extracting only top hit for each sequence^42^. The transcripts were distributed in different groups according to corresponding expression profiles based on abundance in specific pairwise combination. The abundance was then plotted for comparison of leaf and root tissue specific TFs against four species (Fig. 2).

With this approach of TF analysis, we found 93 transcripts in leaf and 46 in root while comparing against *A. thaliana*. Against 922 protein sequences for *C. annum*, 138 leaf transcripts and 97 root transcripts belonging to 36 different families were identified (Fig. 2). Similarly, for 820 protein sequences in case of *N. tabacum*, 62 leaf transcripts and 43 root transcripts belonging to 24 different families were identified. In case of *S. lycopersicum*, 156 leaf transcripts and 107 root transcripts belonging to 37 different families were identified while searching against 1845 protein sequences. However, previously on the basis of annotated data we found 1737 transcripts encoding for transcription factor genes in leaf and 1795 transcripts in root tissues. When the two transcriptomes were blasted against *Arabidopsis* TFs, it was found that GRAS transcription factor was of major occurrence followed by bZIP and C3H transcription factors in leaf and root both; although the proportion of match was greater in leaf. Additionally, HD-ZIP was found to match significantly and exclusively with leaf. Number of transcripts for EIL, TRIHELIX and BEX were similar and close to similar (MYB) in both the tissues. WRKY, TALE, BHLH were having abundant transcript count in root however, as compared to leaf, opposite to CAMTA in which the count in leaf was greater (Fig. 2a). For AP2 the corresponding matching transcripts were not found against *C. annum* although present in the other three species including *S. lycopersicum*, *N. tabacum* and *A. thaliana* with maximum number of matching transcripts observed in leaf tissues when searched against *A. thaliana*. Against *S. lycopersicum* transcripts corresponding to AP2 were found in leaf only, as against *N. tabacum* in which it was found in both the tissues, whereas against *C. annum* no matching homologues were found. In case of ARF *C. annum* consists of matching transcripts in both the tissues, abundance being larger in leaf (Fig. 2a–d). *A. thaliana* and *S. lycopersicum* have shown similar pattern of presence for ARF in leaf only whereas no matching homologues were present in *N. tabacum*. Matching transcripts for ARR-B in both tissues was found in *S. lycopersicum*, number being greater in leaf, whereas in *C. annum* and *N. tabacum* only transcripts corresponding to leaf were found and no matching transcripts were found in *A. thaliana* for this gene. Further B3 matching transcripts were found only in leaf tissues of a *C. annum* and *S. lycopersicum* whereas no similar transcripts were present in other species. BBR-BPC was found in both the tissues when compared with three Solanaceae species while its matching homologue transcripts were absent in *A. thaliana*. Matching transcripts for BES1 gene were absent in *N. tabacum*, found in leaf as well as root tissues of *C. annum* and *A. thaliana* and only in leaf of *S. lycopersicum* (Fig. 2a–d).

Transcripts corresponding to TFs like BZIP, BHLH, C3H, GRAS, TRIHELIX and WRKY were found in both the tissues against all the four species indicating their common occurrence. It may be inferred that these genes may have common pattern of occurrence in most of the plant species^44,48^. On the basis of abundance of transcripts in case of WRKY gene greater number of matching transcripts were observed in roots in *C. annum*, *S. lycopersicum* and *A. thaliana* as opposed to *N. tabacum* in which opposite pattern was found. Similarly, for Trihelix similar abundance was present when compared with *C. annum* and *A. thaliana* and greater number of transcripts for leaf were present as compared to root in *N. tabacum* and *S. lycopersicum*. Moreover, maximum numbers of matched transcripts against the compared species in both the tissues were found in GRAS with same pattern of abundance which was greater in leaf. BZIP has also shown a similar pattern in all four species and have shown a significant occurrence in all four species. For BHLH equal expression of transcripts in case of *S. lycopersicum* was found whereas greater occurrence pattern in root was observed in *N. tabacum* and *A. thaliana* as against leaf and an opposite pattern was observed in case of *C. annum* with leaf abundance being greater. Similarly, for C3H common occurrence pattern was observed against *N. tabacum*, *S. lycopersicum* and *A. thaliana* with leaf abundance being greater and opposite pattern was observed in *C. annum*. No matches for CAMTA gene were present in *N. tabacum* although corresponding results were observed in other species with similar occurrence pattern. *W.somnifera* transcripts for EIL were equally found in root and leaf in case of *A. thaliana* whereas greater leaf transcript occurrence as compared to root was found in studied species of Solanaceae. Presence of transcripts corresponding to transcription factors namely ARR-B, CO-like, DBB, ERF, G2-like, GATA, HB-other, NAC, NF-YC, NIN-like were only limited to Solanaceae member in our study with maximum abundance of transcripts observed against NAC transcription factor. Also, transcripts corresponding to E2F-DP and TCP were restricted to *S. lycopersicum* and *C. annum* only. DOF, FAR1, NF-X1, RAV, VOZ were amongst the gene for which matches were observed in two of the three Solanaceae members in one or both tissues (Fig. 2a–d).

### Top-hit species distribution and study of selected transcription factors

**Figure 3:**
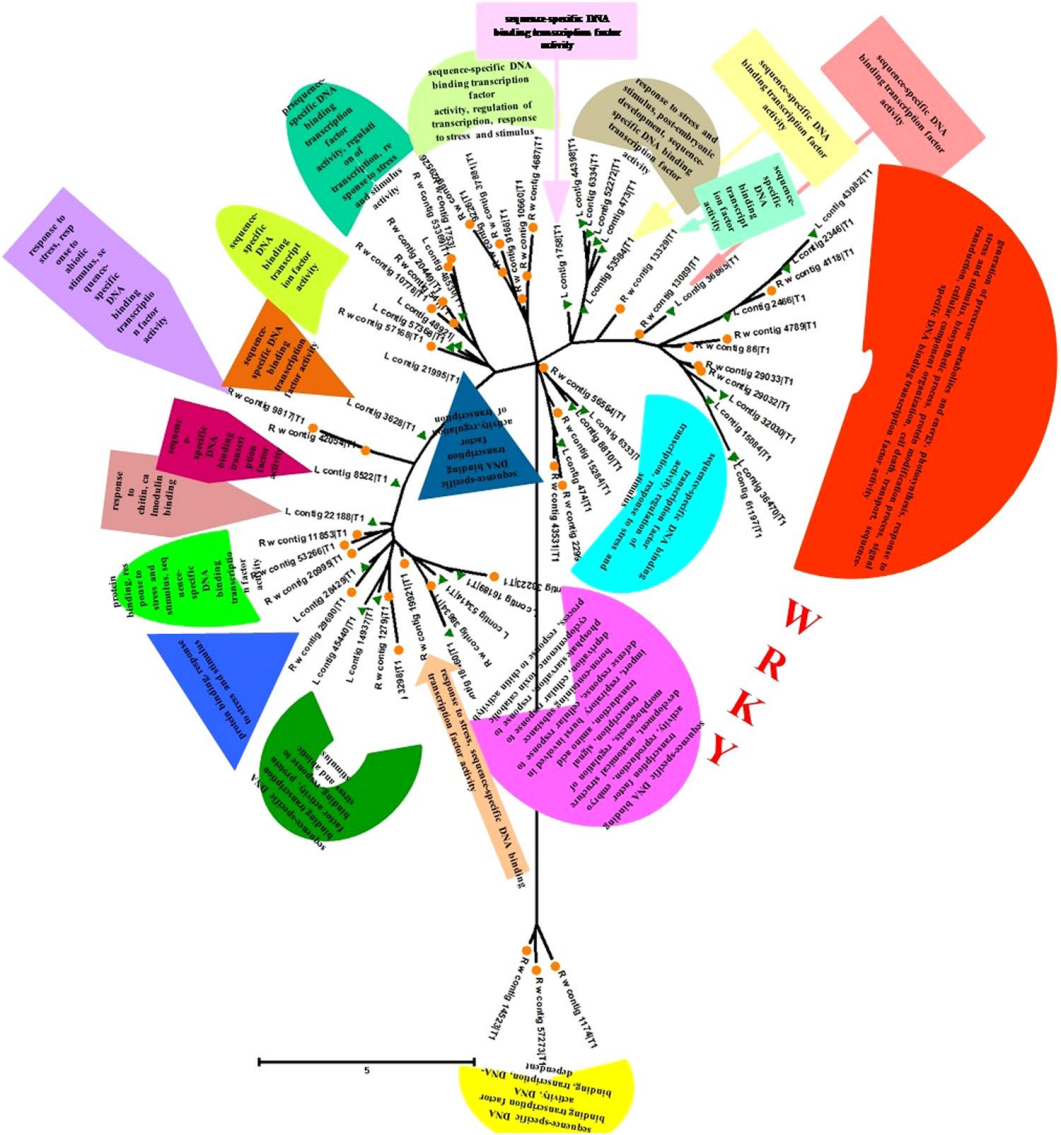
Transcript wise distribution of WRKY family transcription factors. Gene transcripts were classified on the basis of GO annotations.

**Figure 4:**
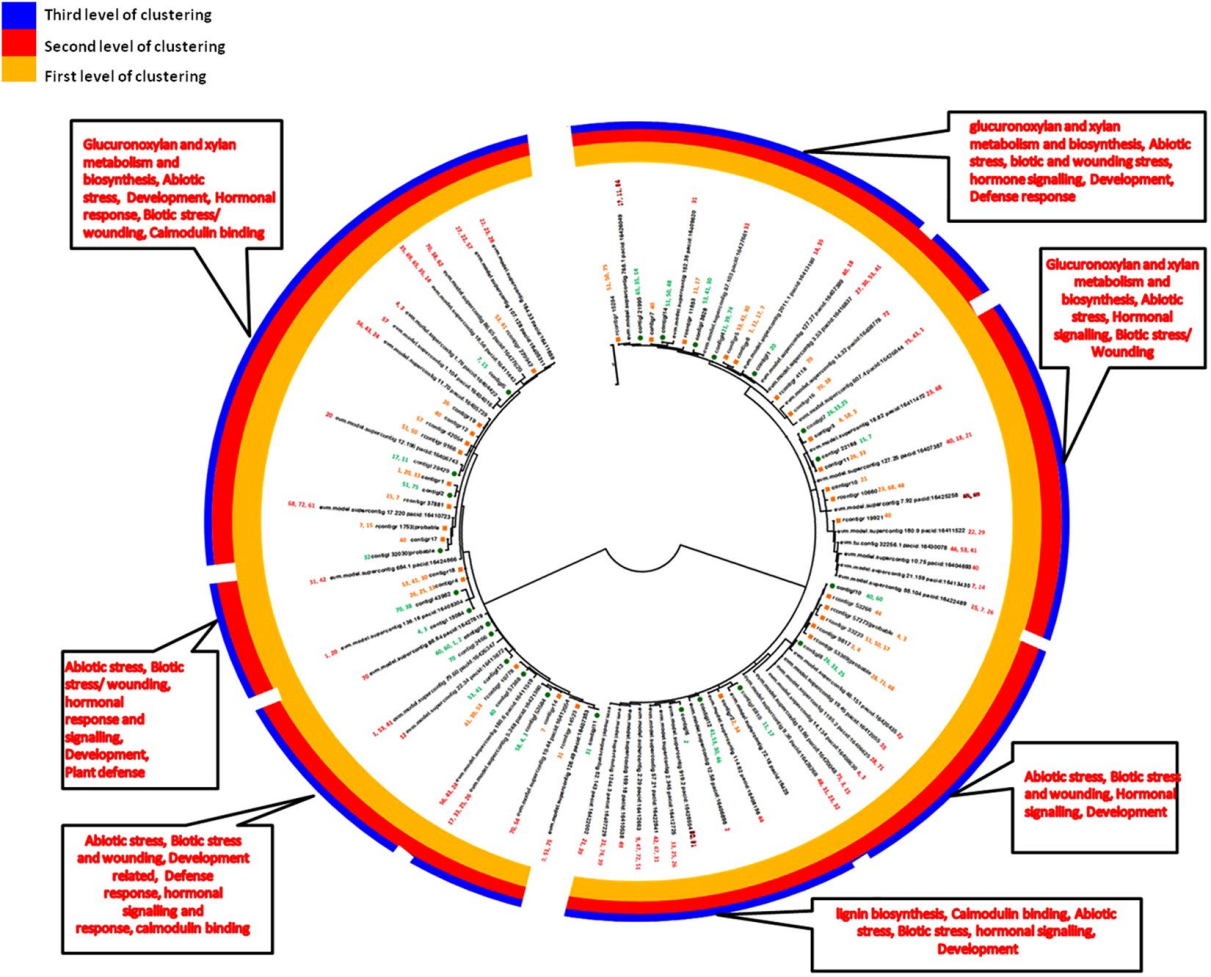
Assignment of WRKY transcription factors to different functions and process. Clustering of WRKY gene transcripts was done based on comparison with *V. Vinifera* WRKY transcripts.

**Figure 5:**
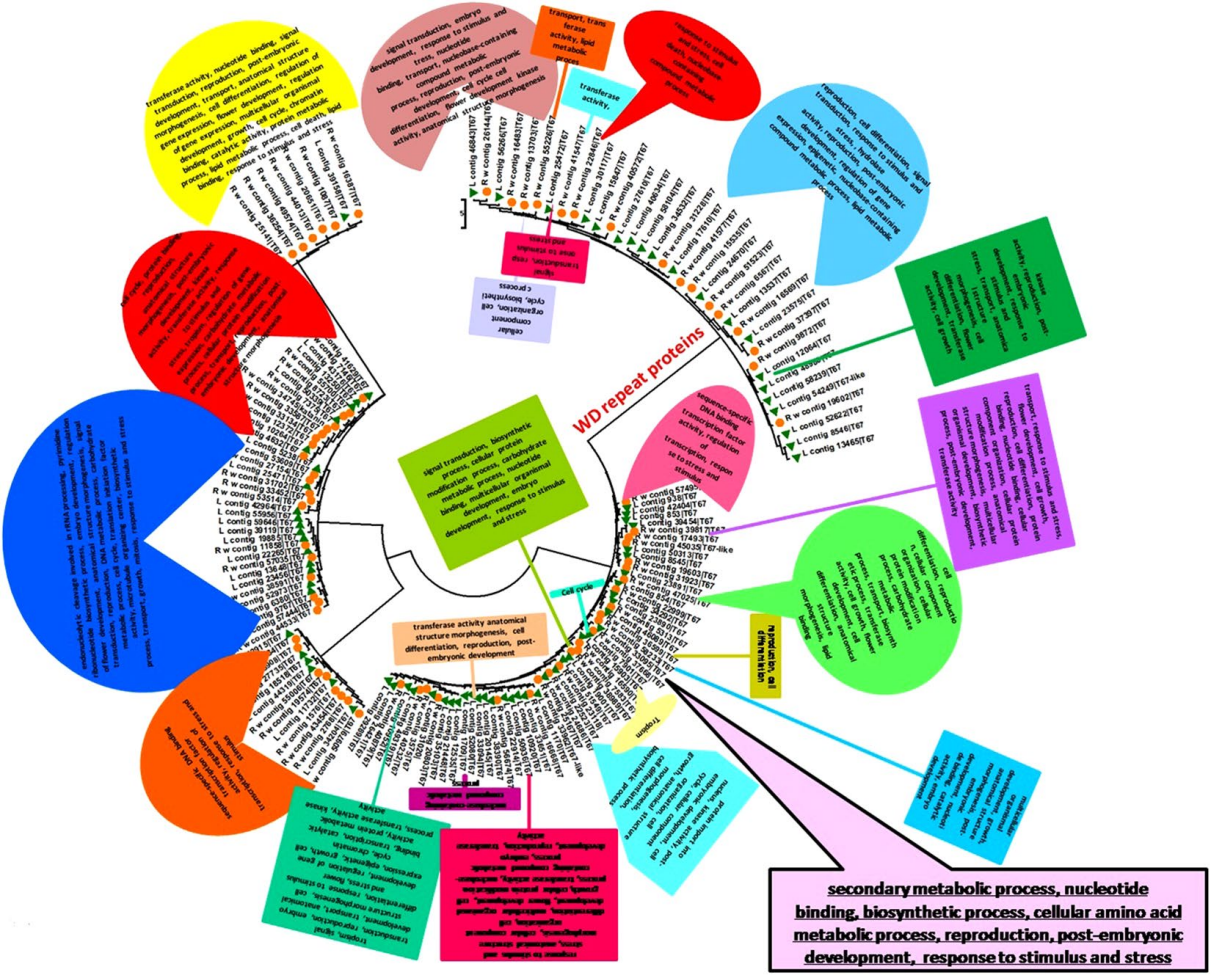
Transcript wise distribution of WDR family transcription factors. Gene transcripts were classified on the basis of GO annotations.

**Figure 6:**
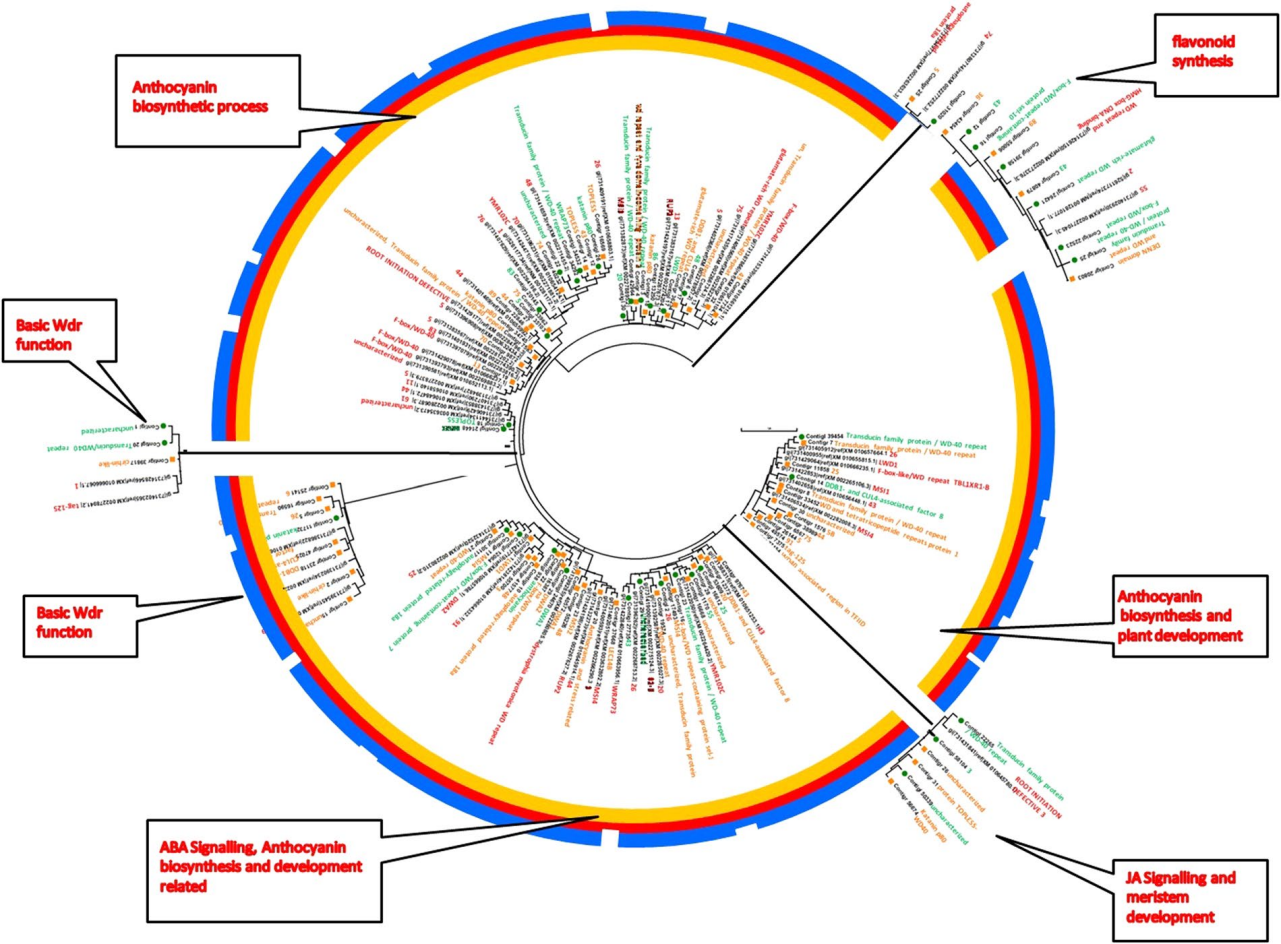
Assignment of WDR transcription factors to different functions and processes. Clustering of *WDR* gene transcripts was done based on comparison with *V. Vinifera* *WDR* transcripts.

In terms of species distribution top hits were obtained for *V. vinifera* in both leaf and root data. However, afterwards the similarity pattern was somewhat different for both i.e in leaf it was followed by *S. lycopersicum*, *P. trichocarpa*, *R. communis*, *G. max*, *N. tabacum*, *S. tuberosum*, *C. annuum*, *M. truncatula*, *N. benthamania* and so on. Similarly, in root tissues, *S. tuberosum* produced second most abundant hits followed by *P. trichocarpa* and then *S. lycopersicum*,. *N. tabacum*, *R. communis* and *G. max* (Supplementary Figure 1). Since the major transcription related transcripts in the two transcriptomes were showing maximum similarity against *V. vinifera*, we clustered the transcription factors of interest namely, WRKY and WDR transcription factors, with *V. vinifera* and also grouped combined transcripts for both leaf and root according to different biological processes and functions. Detailed spectrum of *W.somnifera* TFs and associated biological roles in the comparative analysis had provided distribution of the TFs in three known categories as biological process (BP), molecular function (MF) and cellular component (CC) (Table S2, Figs 3,4,5,6).

### Transcript distribution of WRKY transcription factors with assignment to different functions and processes

Comparison of transcripts in our dataset with transcripts from *V. vinifera* have divided our dataset broadly into two main clades which are further divided into subgroup clades based on the similarity of domains. All the transcripts of *W.somnifera* and contigs from *V. Vinifera* related to WRKY transcription factors were majorly found to be involved in processes like glucuronoxylan and xylan metabolism and biosynthesis, abiotic stress, biotic and wounding stress, hormonal signalling, development and defense response (Fig. 3, Table S3). Some subclades were additionally involved in functions like calmodulin binding, a subclade was also found to be distinctly involved in lignin biosynthesis. Although a common distribution pattern of functions and processes was found amongst the transcripts still they were differently plotted into two different clades and subclades. Root contigs number 15284, 7, 11853, 5, 8 and leaf contigs number 21995, 14, 3626, 4 and 1 were grouped with the genes having functions like drought and salt stress, cold heat and oxidative stress, biotic stress and hormonal signalling, pollen and embryo development, biosynthetic processes like glucuronoxylan and xylan biosynthesis, wounding response to bacteria, seed dormancy, gibberellin response mediated signalling etc. Extended branch to this domain contained root contigs 4118 and 16 grouped with the genes involved in processes like oxidative stress, phosphate limitation, developmental processes, auxin and cytokinin hormonal response along with sugar sensing and metabolism (Fig. 3, Table S3).

The next neighboring subclade contained leaf contigs 7, 22188 and root contigs 3, 11, 10, 10660 and 19921 grouped with genes having involvement in oxidative and drought stress, auxin and cytokinin signalling, salt, cold and heat stress, biotic stress, wounding stress, sugar sensing and metabolism, developmental processes like seed dormancy, positive regulation of plant thermotolerance and ethylene responsive signal transduction pathway. Similarly, the next subclade having leaf contigs 10 and 8 and root contigs 53266, 57273, 33223, 9817 and 53369 were significantly grouped in processes like oxidative stress, drought and salt stress, phosphate limitation, biotic and wounding stress, auxin and cytokinin hormonal signalling, sugar sensing and metabolism, cold stress and gibberellin mediated metabolic processes. In continuation with this the next subclade was having leaf contig 8810 and root contig 2 which were placed close to the genes playing roles in hormonal signalling, lignin biosynthesis and developmental process (Fig. 4).

Now the differently classified clade in Fig. 4 contained root contig 229942 and leaf contig 5 which were presumed to be associated with embryo development leading to seed dormancy, sugar sensing metabolism, pollen development, glucuronoxylan metabolic process and, xylan biosynthetic process etc. Additional involvement was observed in response to chitin, calmodulin binding, cold, oxidative, drought and salt stress, developmental processes, auxin and cytokinin hormonal signaling and gibberellin mediated signal transduction along with bacterial response being categorized with genes involved in above processes. Similarly, root contigs 19, 12, 42054 and 9166 were placed close to *V. vinifera* genes involved in biotic stress and hormonal signaling (Figs 3 and 4, Table S3).

### Transcript distribution of WDR transcription factors with assignment to different functions and processes

Similarly, in *WDR* proteins, some of the transcripts were observed to be associated with secondary metabolic processes, nucleotide binding, biosynthetic process, and cellular amino acid metabolic process. The most frequently occurring terms were tropism, embryo development, flower development regulation, morphogenesis of anatomical structure, signal transduction, reproduction, translation initiation factor activity etc (Fig. 5, Table S4). Some other terms were transport, growth, response to stimulus and stress, epigenetic, nucleobase-containing compound metabolic process, transferase activity, kinase activity, chromatin binding, catalytic activity, protein metabolic process, lipid metabolic process, cell death, lipid binding etc. Some of the terms like endonucleolytic cleavage involved in rRNA processing, pyrimidine ribonucleotide biosynthetic process, microtubule organizing center, mitosis, etc. occurred in a large fraction of transcripts. The term hydrolase activity also occurred in some of the transcripts (Fig. 5, Table S4). When WDR transcripts were compared with *V. vinifera* *WDR* genes, the transcripts were grouped into seven divisions in which three were supposed to be more distantly related and one of the group of sequences was differently classified. The transcripts in the first clade were classified mainly with the genes involved in RNA degradation, in vesicle recycling, biological rythms, histone methylation, phosphate starvation response, actin cytoskeleton organization etc. Major transcripts in this group were contigr 19, 9872, 7583, 74845, 22846 and 27 (Fig. 5, Table S4).

DDB1 and CUL4 is involved in function recognition of ligase complex and is required for plant embryogenesis and further known to affect development of leaf, shoot, and flower in plant. Further this clade was divided into two subclades containing leaf contig 20145, 22,34292, 26 and root contigs 74, 14, 12 and 16569 and it was observed that most of the root and leaf transcripts were also involved in processes like cell cycle, cell division, methylation, rRNA processing, cytoplasm, ribosome biogenesis, autophagy, ubiquitination, methylation etc. It was also found that some contigs like leaf contig 18 and 17 were involved in jasmonic acid signalling pathway and anthocyanin containing compound biosynthetic process. It may be speculated that most of the transcripts were categorized with genes involved in nuclear activities like sister chromatid cohesion, mRNA export from nucleus, protein ubiquitination, regulation of cell cycle process, RNA methylation, rRNA processing etc. These may be further involved in jasmonic acid signalling pathway, anthocyanin biosynthesis and other development related processes like photoperiodic flowering (Fig. 6).

The major reference *WDR* genes for this clade were *WDR*55, *SDR*2, *HMG* box DNA –binding 74 and autophagy related, mainly involved in processes like plant development and flavonoid metabolism in addition to autophagy related processes and ribosome biogenesis. Further the leaf and root contigs in this clade were mainly related to growth and development, general WDR characteristics etc. The next clade was speculated to be involved in anthocyanin biosynthetic process, plant development including floral transition through cold-response genes, metal ion binding and basic characteristic WDR properties. Included root contigs were 27154, 7375, 49574, 26144, 6567, 38989, 1576 and 30 categorized with cold-responsive genes, metal ion binding and plant development related genes through activities at chromatin level. Functions may be assigned to some of the transcripts for which no function is known. Further, one of the reference genes in this clade is also found to be involved in anthocyanin biosynthetic process (Figs 5 and 6, Table S4).

A clade with distantly plotted branch was categorized near to the previous clade contain reference protein root initiation defective 3 (RID3) which is basically involved in meristems development and function as a negative regulator of shoot apical meristem (SAM) neo-formation through CUC-STM pathway. Further the contigs from root and leaf tissues were supposed to be linked with jasmonic acid signaling pathway and development related process in addition to the basic functions carried out by WDR proteins (Figs 5 and 6, Table S4).

Next to this another larger clade divided into two subclades was found and major reference proteins in this clade were related with ABA signaling pathway, plant development involving photoperiodic flowering cold responsive and metal ion binding in addition to the basic WDR gene functions like ubiquitination, ribosome biogenesis, vesicle recycling etc. The first subclade mainly contains genes with basic WDR protein function and development related functions as well as genes with unknown functions (Figs 5 and 6, Table S4). The major contigs for leaf were 16612, 2329, 27735 and root were 9767, 12372, 53609, 28, 16668, 16, 17493 and 19574. The other subclade contains the contigs and genes mainly related with the processes like fruit ripening, anthocyanin and stress (cold responsive) related ABA signaling, transport metal ion binding, development related in addition to characteristics WDR functions like chromatin modelling, mRNA processing splicing, vesicle recycling etc. Definitions of some of the WDR entities like WD repeat 25, 91 and related transcripts were however found to be unknown. The root contigs involved were contigr 37668, contigr 20, contigr 23, contigr 55226, contigr 40572, contigr 29, contigr 22, contigr 10, contigr 41557, contigr 55700, contigr 1, contigr 11 and contigr 21. In case of leaf major transcripts were contigl 1, contigl 938, contigl 12064, contig l 30117. It was noticeable that first group contains only the contigs from root (Figs 5 and 6, Table S4). The positions of the transcripts in the remaining subgroup is reliable as most of the transcripts lie close to the similarly annotated genes as evident by anthocyanin related transcripts DWA related, MSI related, WDR 43 and unknown functions. Next to this the differently placed clade majorly grouped root contigs with one leaf contig 11732, katanin p80, WD 40 along with proteins related to development through mRNA surveillance pathway. Similarly, the next distantly placed smaller clade contains the protein sequences and transcripts involved in processes like histone modification proteins, actin cytoskeleton organization, and other basic WDR functions. In this group cirrhin like protein 4 was also present which functions as U3 small nucleolar RNA associated molecular scaffolds (Figs 5 and 6).

### Transcripts abundance of *WsLWD1* and *WsWUSCHEL* TFs in *W. somnifera* tissues

**Figure 7:**
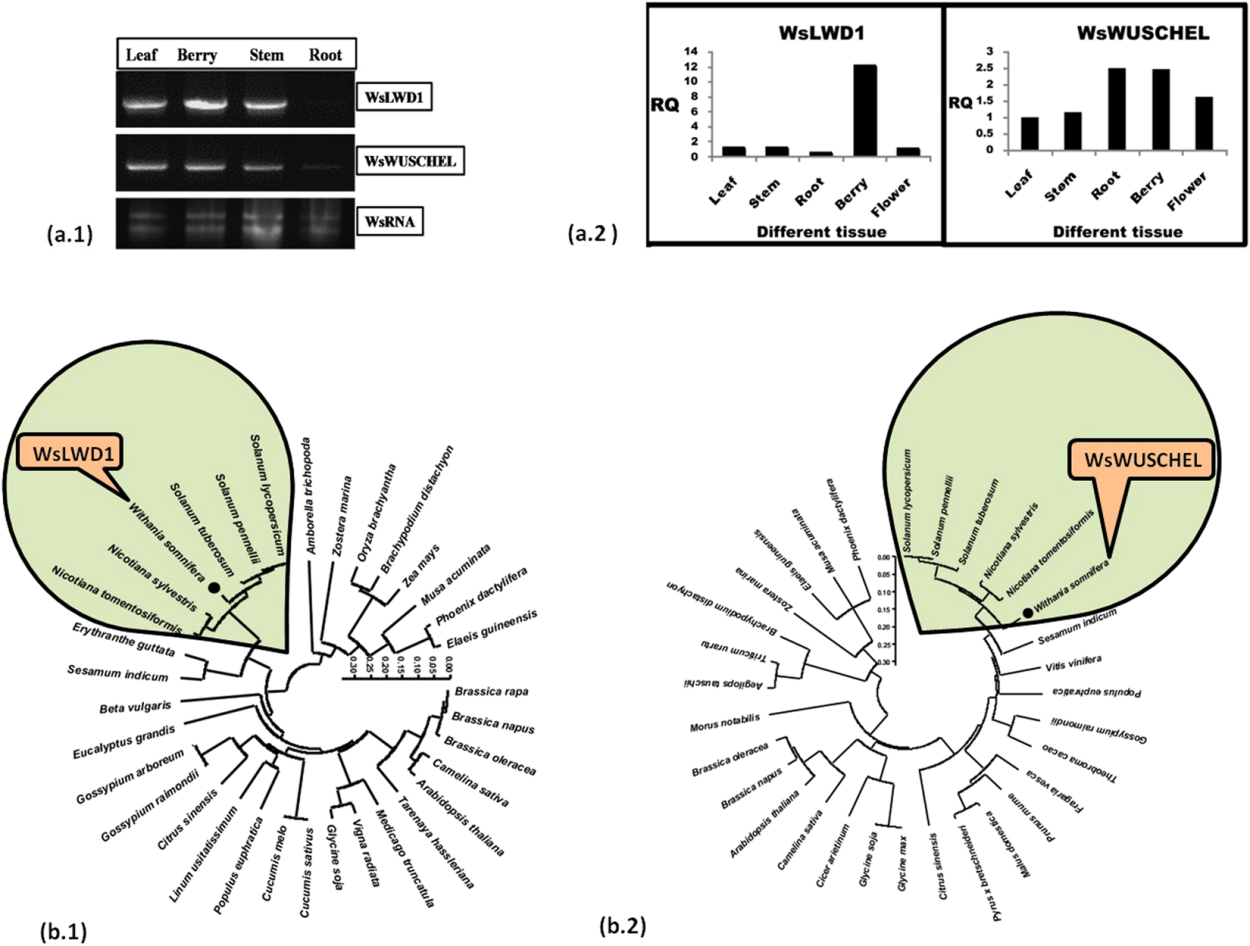
Lab validation of full length TF gene transcripts. **(a**) Representative of full length transcript from two transcripts annotated as *WsLWD1* and *WsWUSCHEL* from WDR gene family were subjected to validation in lab. (**a.1)** Semi quantitative analysis of WDR (*WsLWD1* and *WsWUSCHEL*) full length transcripts using cDNA transcribed from *W.somnifera* RNA (WsRNA). (**a.2)** Quantitative expression analysis of the respective transcription factors in various tissues of *W.somnifera*.**(b)** Phylogenetic analysis of full length protein of the respective genes in *W. somnifera* **(b.1)** *WsLWD*1 transcription factor and **(b.2)** *WsWUSCHEL* related homeobox 11 transcription factor using the maximum likelihood method through MEGA 6.06 tool.

After identification of overall transcription factor repertoire, we examined specific families whose transcripts were having full length coding regions. (Fig. 7a.1, a.2). Both the transcription factors were expressed in all the tissues but their abundance was quite elevated in berry and very less in root tissues. *WsLWD1* have shown maximum expression in berry followed by leaf and stem and then flower having minimum expression in root. *WsWUSCHEL* also shown higher expression in root as well as in berry followed by leaf and stem.

### Full length sequence isolation and analysis of WDR transcription factors (*WsLWD1* and *WsWUSCHEL*)

**Figure 8:**
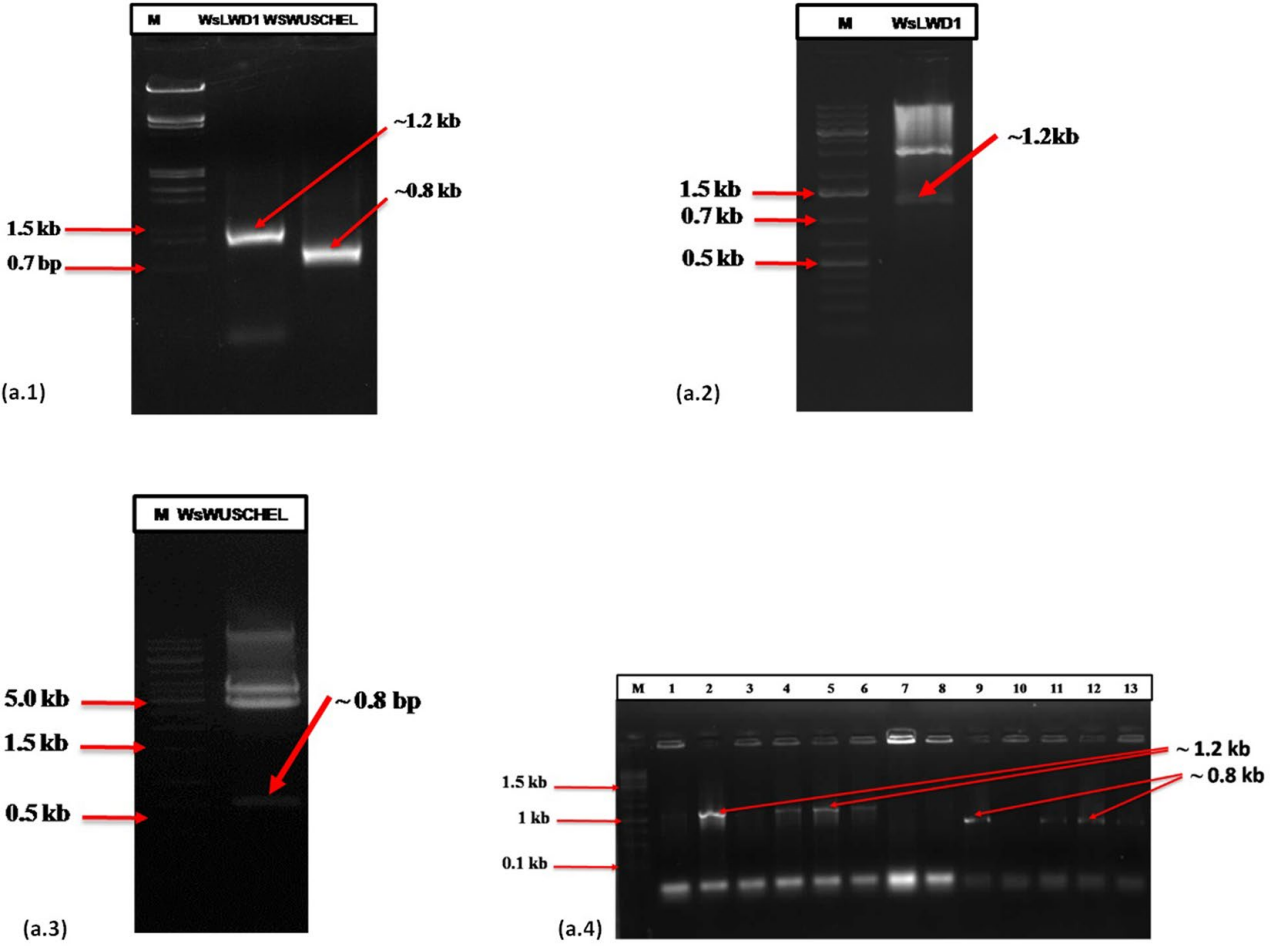
Restriction digestion and colony pcr confirmation of full length genes. (**a.1)** Amplification of WDRs**-** *WsLWD1*(1.2 Kb) and *WsWUSCHEL* related homeobox 11b (0.8 Kb) **(a.2** and **a.3)** Restriction digestion of TFs by respective restriction enzymes and **(a.4)** Colony PCR confirmation of the two genes (*WsLWD* 1-7 and *WsWUSCHEL* 8-13) cloned in pBI121 plant vector.

The full length *WsLWD1*contained 1131 bp and *WsWUSCHEL* contained 786 bp nucleotide sequences encoding 377 and 262 amino acid protein respectively. BlastX analysis of *WsLWD1*and *WsWUSCHEL* revealed that they are related to WDRLWD1/THO complex subunit 6 and WUSCHEL associated homeobox 11. To confirm the assembly and further wet lab analysis full length sequences for *WsLWD1* and *WsWUSCHEL*, corresponding genes were isolated from cDNA library of *W. somnifera* fruit tissues and cloned. These cloned plasmids of *WsLWD1* and *WsWUSCHEL* were sequenced and confirmed by BlastX search as WDR LWD1 and WUSCHEL related homeobox 11 transcription factor (Fig. 7a,b, Fig. 8). Full length *WsLWD1* have shown homology with other WDR LWD1 transcription factors as *S. tuberosum* (92%), *S. lycopersicum* (91%) *N. tomentosiformis* (89%), *S. indicum* (69%) *G. raimondii* (67%) *P. euphratica* (66%) *C. sinensis* (66%) *M. truncatula* (63%) and *A. thaliana* (58%). Full length *WsWUSCHEL* was showing homology with other WUSCHEL related homeobox 11 transcription factors as *N. tomentosiformis* (75%), *N. sylvestris* (73%) *S. lycopersicum* (78%), *S. tuberosum* (71%), *T. cacao* (62%), *C. sinensis* (60%), *M. domestica* (57%) and *A. thaliana* (57%). The theoretical pI of *WsLWD1* and *WsWUSCHEL* proteins were 4.80 & 6.44 and their molecular weight were 40.92 & 27.47 kDa. Phylogenetic analysis of WDR *WsLWD1* revealed that it is closely related to *S. tuberosum, S. pennellii, S. lycopersicum, N. tomentosiformis* and *N. sylvestris* which comes under same family Solanaceae as *W. somnifera*. Similar results were obtained for *WsWUSCHEL* related homeobox 11 transcription factor, which falls in groups of plants such as *N. tomentosiformis, N. sylvestris S. tuberosum, S. pennellii* and *S. lycopersicum*. (Fig. 7b.1 and b.2).

### Validation of abundance of *WDR* transcription factors (*WsLWD1* and *WsWUSCHEL*) through *in planta* transformation and metabolite estimation

**Figure 9:**
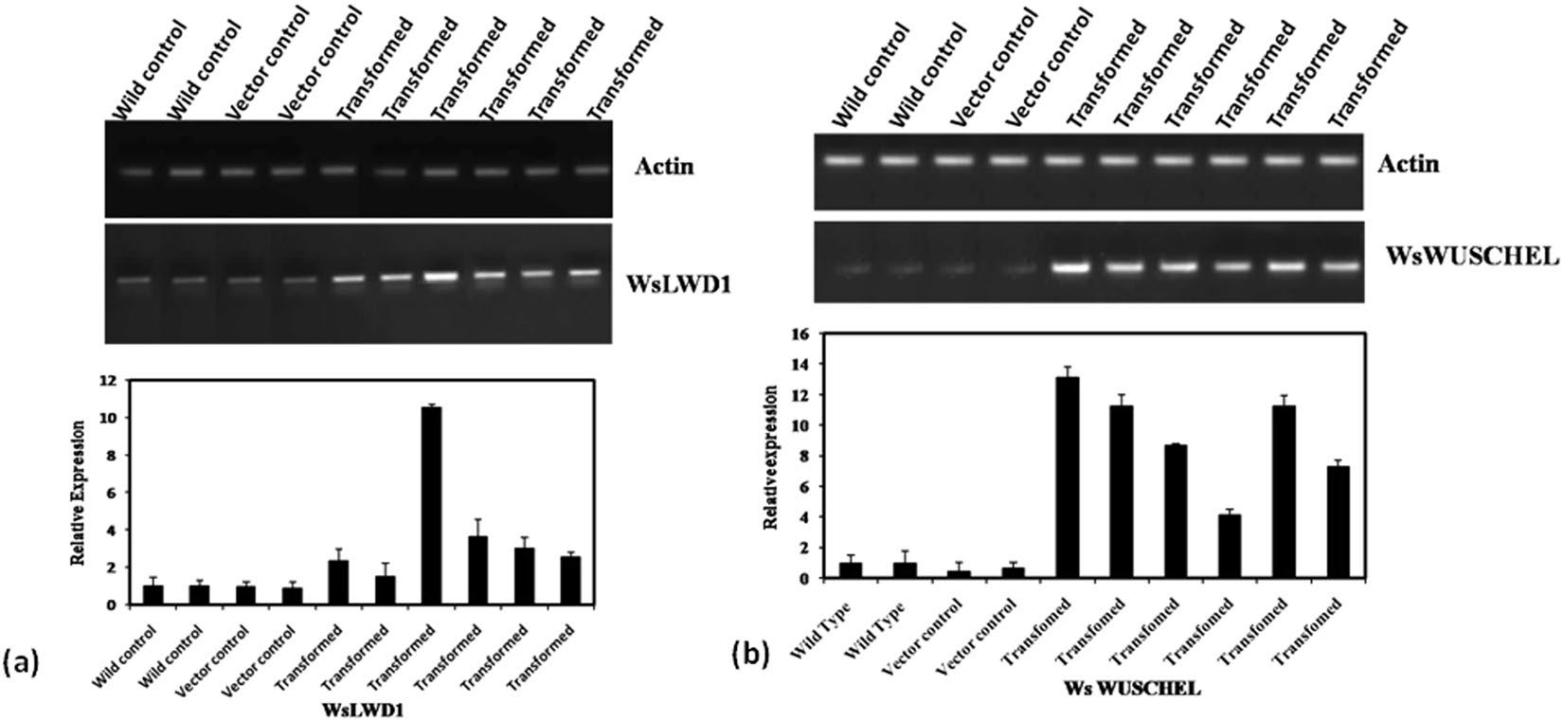
Gene expression analysis of transcription factors (*WsLWD1*and *WsWUSCHEL*) in transiently transformed *W. somnifera* leaves. **(a)** Semi-quantitative and real time qRT PCR analysis of *WsLWD1* gene in wild type, vector control and transiently transformed leaves of *W.somnifera*. **(b)** Semi-quantitative and real time qRT PCR analysis of *WsWUSCHEL* gene in wild type, vector control and transiently transformed leaves of *W.somnifera*.

*WsWUSCHEL* and *WsLWD1* TF genes were further used for transient expression and semi-quantitative as well as quantitative PCR was again performed to ascertain their expression in transiently overexpressing lines of *W. somnifera* in comparison with wild type and empty vector control. Transcript levels of *WsWUSCHEL* and *WsLWD1*genes were highly up regulated in transiently transformed lines in comparison to wild and empty vector control samples. This was also in correlation with real time analysis of the samples. The results demonstrated that selected transcripts have shown upregulation in overexpressing lines. Quantitative real time PCR was done to quantify the transcript (expression) of *WsWUSCHEL* and *WsLWD1* transcription factor genes. The data showed elevated pattern in transcripts of *WsLWD1* and *WsWUSCHEL*, genes in the transgenic lines, though expression of these genes varied among individual lines. In transiently transformed lines of *WsLWD1* the expression was upregulated from 1.5 fold to 3 fold in comparison to wild type explants and empty vector control explants (Fig. 9a). In transiently transformed tissues of *W. somnifera* overexpression of *WsWUSCHEL* gene was 4 to 13 times higher in comparison to wild type explants and empty vector control explants (Fig. 9b).

**Figure 10:**
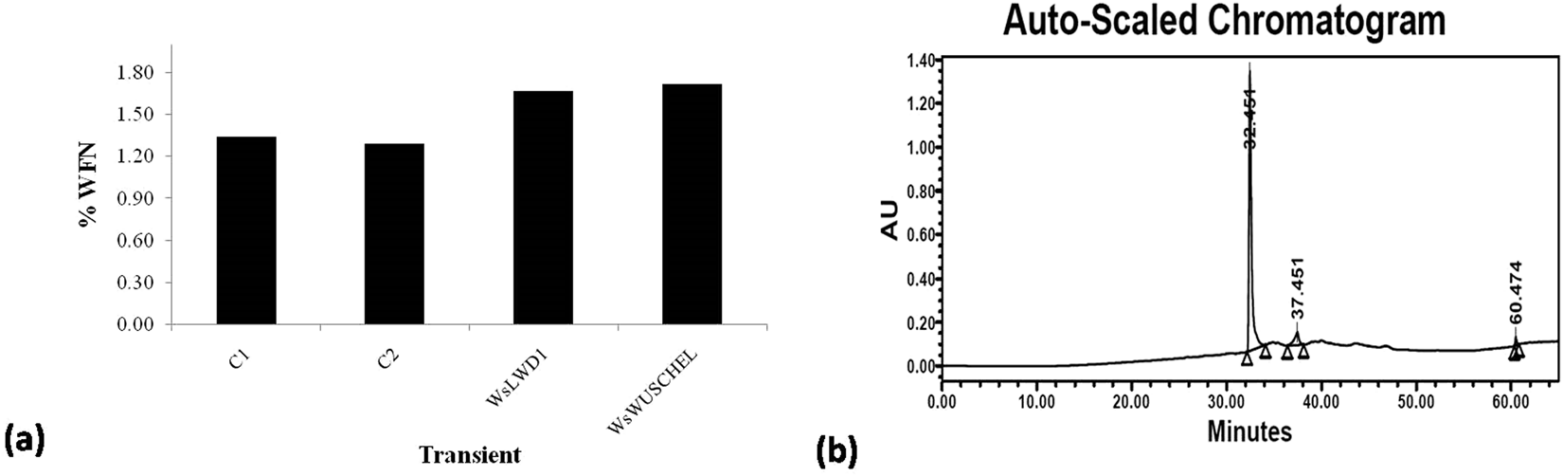
Withanolide content in *W. somnifera* leaves transiently transformed with *WsLWD1* and *WsWUSCHEL* TFs **(a)** Withanolide content expressed as withaferin A in transiently  transformed tissues of W. somnifera with WDR genes  (*WsLWD1* and *WsWUSCHEL*). **(b)** Representative  HPLC-chromatogram of  the *W. somnifera* tissue extract.

Further, to evaluate the role of these transcription factor genes in withanolide accumulation, we estimated withanolides from individual lines and quantified^21,40^. Withanolide analyses also revealed that withanolide accumulation was upregulated in the transiently transformed tissues in comparison to control tissues (Fig. 6 a, b).Withanolide analysis of all the transient lines revealed that *WsLWD1* and *WsWUSCHEL* might be linked with withanolide biosynthesis as it is affecting the withanolide biosynthesis. Further, the percentage of withaferin A (WFN) was higher in *WsLWD1*and *WsWUSCHEL* transient lines in comparison to control (Fig. 10).

## Discussion

The results of present study on analysis of transcriptomic sequences from TF population and functions in *Withania somnifera* have revealed role and significance of regulatory transcription factors with respect to different processes including biosynthesis of withanolides, the characteristic specialized metabolites of the medicinal herb in a tissue-specific manner^1–3^. TF dataset generated here presents important tools and targets for advancement of knowledge on regulatory genetic elements including novel functions with respect to action and/or tissue-specific function through their differential expression pattern in different tissues^4,5^. Thus, the results represent a detailed recognition of TF-encoding transcripts from the transcriptomic database resources available for *W.somnifera*, as add on to and expansion of earlier studies^9,10^. The results provide a comprehensive picture of repertoire of regulatory factors of this important medicinal plant^1–3^ (Fig. 1, Table S2). TF-encoding transcripts set based sequence similarity with known transcription factor genes using state of the art analytic parameters have led to higher level of forecast along with assignment to functions and conserved domains which guided clustering with known structural families^41^ (Figs 3,4,5,6). The results revealed abundance of transcripts for WDR and WRKY transcription factors in the two tissues and suggested their important roles as a part of regulatory mechanism for metabolic processes in *W. somnifera* ^2^ (Figs 3,4,5,6 Tables S3,S4). The next level of abundance observed for FBOX, BZIP, BHLH, GRAS and AP2 related transcription factors in the two tissues indicated their involvement in various biological processes of the plant (Fig. 1, TFs under study, data unpublished)^2^. The comparative analysis of the transcriptomic sequences from the two tissues revealed differential expression of transcription factor gene families (Fig. 2). It appears to be emphatic to secondary metabolite biosynthesis and accumulation guided by species identity, tissue-specificity as well as tight spatio-temporal regulation of gene expression^43^. There may be variable expression of TF gene family in different plants at least for some, though others were highly specific in their presence as well as absence^1–5,42,43^. In response to different spatio-temporal conditions, differential expression of similar TF genes could result due to *cis*-acting elements^3^. This signifies that the regulation of gene expression along with function of a particular TF is the result of vital network of several interrelated processes^2^ (Table S2). PlantTFDB represents an integrated collection of complete TF sequence sets in genomes of different plant species for sequence retrieval and complete annotation^42^. To explore specific TFs we performed extensive, comprehensive and comparative TF analysis between the closely related species as there may be species-specific adaptation for a particular TF gene family^1–5,43^. Some of the transcription factors like BHLH, C3H, GRAS etc. were abundantly found in all species sampled in the comparison suggesting the important role in plant metabolism whereas more abundance of certain nature of transcription factors like MYB, NAC and WRKY reflected a case for Solanaceae family^2^, as opposed to *A. thaliana* suggesting the species specific abundance of occurrence of these TFs (Fig. 2). Comparison of the transcriptome with *A. thaliana* and other Solanaceous members provides the rationalized comprehensive view of specificity and ubiquity of different transcription factors across the species compared^2,5,6^. The number of candidate transcripts obtained on manual identification is more as against those determined by comparative search against *A. thaliana* and other species indicating the presence of specific transcription related proteins in *W. somnifera* which were unidentified earlier^2,3^ (Fig. 2). The observed attribute of tissue-specific abundance of expression was interesting. Its functional overtone may be combinatorial programming based recruitment of TFs to dictate transcriptional regulation of different tissues in response to several environmental signals and to serve their respective developmental signs and specificities^3,44^. The observed matching of results from real-time quantitative analyses of TF genes in plant tissues were confirmatory to *in silico* predictions (Fig. 2). In the absence of genome sequences of the plant, bioinformatics based processing of available transcriptomic data proves to be an essential resource to account for key proteins of metabolic network regulation on the basis of expression levels^1–5,44^.

Expression analyses and grouping of transcripts according to GO terms in addition to comparison with *V. vinifera* genes suggests important links for the abundantly present transcription factor genes WRKY and WDR proteins to be involved in biosynthetic route regulation and other important biological processes^2,3,44^. It has been shown recently in many studies that transcription factors contribute strongly in secondary metabolites regulatory mechanism and WRKY^45^ are amongst majorly analyzed proteins in this perspectives in the plants^44^. Further, being sessile, plants continuously suffers from various types of environmental stresses like drought, cold, salinity, nutrient starvation etc^35^ (Figs 3,4,5,6 Tables S3, S4). In response to stresses, plants are either stress tolerant or stress responsive^35^. Stress related genes are induced at transcriptional level^2^. WRKY proteins are involved in different types of biotic as well as abiotic stresses in addition to other physiological processes embryogenesis, leaf senescence, hormone signaling, seed coat and trichome morphogenesis^45–48^. Besides these roles, WRKY proteins are reported to be associated with regulation of biosynthesis of different secondary metabolites synthesized in a number of plants^48^. However, hardly any deep study has yet been performed to find the potential role of WRKYs in regulatory mechanism of secondary metabolites in the perspective of physiological function of the specialized phytochemicals^45^. Many gaps still exists concerned to the network explaining the regulatory mechanism of metabolites accumulation through these genes in plant cell. WRKY family of proteins contains one or more extremely conserved heptapeptide WRKYGQK domain accompanied with a zinc-finger motif required for the modulation of gene expression (Figs 3,4 and Table S3). This WRKY domain attaches to W-BOX element present in the promoter of a target gene^49,50^. A WRKY gene has also reported from *P. quinquefolius* that play important role in ginsenoside production^49^. Chemically, withanolides could be compared with ginsosides which represents secondary metabolites from *P. quinquefolius*, American ginseng, both being of triterpenoid origin. Therefore, secondary metabolites obtained from plants reportedly have important role in defense related mechanisms of the plant^49,51^. Several inter-connected pathways exist in plant cell which supervise the metabolites flux however, no proper proofs are available describing the proper mechanism of information flow to regulate the related biosynthesis in cell (Figs 3,4 and Table S3). In our study, WRKY related transcripts were broadly found to be involved in glucuronoxylan and xylan biosynthesis, various stress like abiotic/biotic/wounding leading to plant defense mechanism, hormonal and signaling responses in addition to developmental processes etc. on the basis of similarity with *V. vinifera* transcription factors^2,3,44,49^ (Figs 3,4 and Table S3).

WDR, the next major family of TFs recognized in *W. somnifera* in this study is interesting as, WDR family of TF is diversely membered by transcription factors in eukaryotes, which functions as a platforms of protein-protein interactions and having their functional manifestation in a variety of biological processes, such as gene regulation at transcriptional level, signal transduction, modification process of proteins, vesicular trafficking, assembly of cytoskeletal elements, cell death and cell cycle progression^52^ (Figs 5,6 and Table S4). These proteins are also linked with a variety of other processes like transcription regulation to cell cycle control, apoptosis, chromatin assembly, mRNA synthesis, RNA splicing, transcription initiation complex assembly and often organization of protein into complexes^53,54^. The well known characteristic feature of these TFs is a WD motif (Trp-Asp or WD-40) of ~40 amino acid stretch, with limited conservation of amino acid sequences^44,52,55,56^. The characteristic WDR motif of WD protein is GHSDYLHCIVARNSHNQVITGSEDGTARLWD. Only a few reports are available on WDR LWD proteins from plants, especially in *A. thaliana, C. sativus* L, and *L. usitatissimum* L^54,55-58^. By using genetic approaches, a total of 82 *Arabidopsis* WDR proteins have been reported to be involved in plant specific processes^59^. In our study, this transcription factor superfamily was expected to be largely involved in anthocyanin biosynthetic process, ABA, JA signaling and development related process in addition to the basic WDR functions, as inferred from similarity with *V. vinifera* ^52,54^ (Figs 5,6 and Table S4). WDR LWD1 protein in *L. usitatissimum* L. (Flax) regulates pollen growth and viability which provide a new insight in male sterility mechanism present in flax^56^. LWD1 is a clock protein involved in photoperiod regulation of plants. Similarly, WUSCHEL-RELATED HOMEOBOX (WOX) gene family belongs to homeobox (HB) super family which is recognized by a 60-65 amino acid homeodomain. This protein is considered to be involved in specialized functions such as developmental processes comprising stem-cell maintenance, embryonic patterning and organ formation^60,61^. These genes also known to play important role in coordinating gene transcription by being involved in shoot as well as root meristem function and organogenesis (Figs 5,6 and Table S4). The family encodes a homeodomain necessary for the structural integrity and morphogenesis related with shoot and floral meristems^55,60–63^. It maintains the shoot meristem proportion by asserting the pluripotent stem cells number in shoot meristem. There is a feedback mechanism between WUS and CLAVATA protein^64,65^. Mutations in WUS gene have been shown to result in defective inflorescence and vegetative development. Wuschel have also role in adventitious root development^66^. Further middle domain specific Wuschel genes considerably known to be involved in leaf development^53,66^. About 15 members of this WOX family (1 WUS and WOX 1-14) were available in plants, out of them *A. thaliana* possess all the members whilst others like rice, tomato, *Petunia*, maize, snapdragon contain some of them^66,67^. WOX11 protein is involved in auxin as well as cytokinin signaling that regulates proliferation of cells while crown root development occurs and expressed in regions related to division of cells in shoot and root meristems^66,67^. The development of crown root growth was inhibited by silencing of WOX11 while its over-expression led to crown root cell division, accelerated precocious growth and production of crown roots in upper stem nodes^52,68^. Although the above genes have been widely studied with respect to other functions and processes, however, these have not been cloned and their role has not been ascertained in *W. somnifera* ^44,48^. Using transient expression system, that serves as a short-path method of transformation alternative stable transformation for certain^68,69^, it was used to analyze the TF function.

The observed major abundance of WRKY and WDR proteins related transcripts reflected significant role(s) of these two classes of transcription factors in *W. somnifera*. Therefore, these two classes of transcription factor proteins were deeply analyzed in the plant^2,3,45,65^ (Fig. 1). Enriched gene ontology terms were obtained for the two transcription factor groups in our data (Figs 3,4,5,6). On comparison in a species-wide scale, majority of the transcripts were found homologous to *S. lycopersicum* TF repertoire depicting its close relatedness with *W. somnifera* ^2,43^ (Fig. 7b). A major group of transcripts annotated as WRKY transcription factor are observed to be involved in stress driven signal transduction to generate precursors of metabolites and energy through involvement in hormonal signaling and other developmental programs^2,3,45,71^ (Figs 3,4 Table S3). Although, a major proportion of transcripts for WDR proteins in *W. somnifera* was associated with regulation of growth and development related processes^2,3,52^, the transcripts were additionally observed to be associated with secondary metabolism indicating such possible dual role of these regulatory proteins in *W. somnifera* (Figs 5,6 Table S4). Role of WRKY proteins in *W. somnifera* has earlier been established with respect to secondary metabolic pathway related genes^14^, therefore the results from this study are more emphatic to their abundance in numbers and corresponding domain of regulatory effects. However, the study on role of WDR proteins namely *WsLWD1* and *WsWUSCHEL* analyzed *ab initio* for the plant. The observed maximum expression of these TFs of WDR family in berry suggested their association with organ specific growth and development^2,3,14,52,69^(Figs 5,6,7). Tissues transiently transformed with these TFs, showed enhanced withanolide content confirming the additional role of WDR^52–68^ proteins in secondary metabolism^44,48^.Thus, the study demonstrates that the two WDR proteins have regulatory role in both organ development processes as well as secondary metabolite accumulation in *W. somnifera*. Further studies in this regard are underway and will soon provide more elaborate evidence of functioning of other related proteins in *W. somnifera*.

The results on comparison with *A. thaliana* have provided an overview of basic transcription factors which the plant utilizes to adapt itself to the environmental conditions^6,44,48^ whereas comparison with Solanaceous species provided the pathway and metabolite based specificity of respective plant family (Fig. 7). Phylogenetic analysis with similar genes of other plant species has revealed the species-specific evolution of WDR genes as represented by separate clustering of Solanaceae family members^2,52–58^ (Fig. 7a,b and Fig. 8).This could be a sign of representation of regular evolutionary thrust towards biochemical novelty leading to the restriction of major secondary metabolites to constricted phylogenetic lineages in various family, genus or species^2,6,44^.

The results of the study provide a specialized platform for TF genes to help in future studies through construction of a full-scale interactome and elucidation of regulons which control particular TF, for understanding coordination of metabolic processes with cellular and developmental processes at molecular level in Ashwagandha^2,21,43,70^. In the absence of *W. somnifera* genome sequence data, the study is expected to play an essential resource for addressing key regulatory proteins involved in developmental processes and in the biosynthesis of secondary metabolites^2,43^. On the basis of these results we speculate that the we have presented the best possible reports of transcription factor distribution in *Withania somnifera* from the currently available data (Fig. 1, Table S2). On the other hand, with the uprising of greater in-depth and efficient NGS approaches some newer aspects of transcription factor occurrence might get revealed possibly^2,8,43,44^. TF-encoding gene identification offers insights into the organization of TFs in this important medicinal plant^42^. Also, it would provide cues for strategizing genetic engineering of the plant for enhanced metabolite content^69^. Despite the TFs studied and validated through transcriptomic and expression analysis at this scale, it is possible that many other minor TFs may have major role specifically in characteristic metabolite biosynthesis in the plant^43,48^.

In conclusion, as per currently available transcriptomic sequence resources and the level of their analysis for TF mining, WDR and WRKY proteins represent the most abundant group of proteins in *W. somnifera* ^2,43^. The abundant feature of the plant might play some important role affecting plant physiology and adaptive mechanisms towards various environmental conditions^44,48^. The two proteins are reported to have major involvement in developmental processes in which one (LWD1) is involved in regulation of circadian clock, while the other (WUSCHEL) is mainly involved in organogenesis and development related functions^52–68^. As the plant has distribution in varied environmental conditions (including xeric, humid, cold etc.) and geographical regions, possibly the two WDR proteins (LWD1 and WUSCHEL) being abundant have some direct or indirect regulatory roles affecting the physiology and the metabolism of the plant in turn^8,48^. In *Withania somnifera* tight linkage of differential accumulation pattern of major withanolides (i.e. withanolide A, withaferin A alongwith withanone) was shown in various morphogenic stages while the organogenesis occurs^18,34^. In our earlier reports, the effect of correlation between photoperiod, growth, development and secondary metabolite biosynthesis have also been shown^71^. Further, a correlation between secondary metabolism with primary metabolism and development has also been established earlier^72^. In another report, the terpenoid metabolism was shown to be affected by various physiological stages of infloresecence and leaves in *Cymbopogon* ^73^. An interrelation between secondary metabolite biosynthetic process and metabolite content affected with various physiological factors was also shown in a study^74^. Therefore, the results from WDR and WRKY expression *in planta* and that from transient transformation expression studies *vis-à-vis* withanolide levels suggest the possibility of WDR proteins to be involved in secondary metabolite biosynthesis, besides their usual role in other process such as plant development etc. Understanding the regulation through these transcription factors will therefore help in developing the varieties of the plant adapted to diverse climatic conditions, ensure better lines of the varieties in different generations and facilitate identification of the appropriate developmental stage for maximal biosynthesis.

## Electronic supplementary material

Supplementary Information
