# Supplementary material for: Transcription factor repertoire in Ashwagandha (Withania somnifera) through analytics of transcriptomic resources: Insights into regulation of development and withanolide metabolism
Source: Sci Rep. 2017 Nov 30;7:16649. doi: 10.1038/s41598-017-14657-6 (PMC5709440; doi:10.1038/s41598-017-14657-6)
Supplement: Supplementary file 1 — Supplementary Information [file 41598_2017_14657_MOESM1_ESM.pdf]

Supplementary Information

Transcription factor repertoire in Ashwagandha (*Withania somnifera*) through analytics of transcriptomic resources: Insights into regulation of development and withanolide metabolism

Sandhya Tripathi[13], Rajender Singh Sangwan[123], Lokesh Kumar Narnoliya[1], Yashdeep Srivastava[1], Bhawana Mishra[13], Neelam Singh Sangwan[13*]

[1]Department of Metabolic and Structural Biology, CSIR-Central Institute of Medicinal and Aromatic Plants (CSIR-CIMAP), Lucknow 226015, India

[2]Center of Innovative and Applied Bioprocessing (A National Institute under Department of Biotechnology, Govt. of India), Sector-81 Knowledge City, PO Manauli, S.A.S. Nagar, Mohali-140306, Punjab India

[3]Academy of Scientific and Innovative Research (AcSIR) (*An Institution of National Importance by Act of Parliament*) AcSIR Campus Ghaziabad-201002, UP, India

*Corresponding author

E-mail: nsangwan5@gmail.com; Phone no:05222718633; FAX: 0522-23246666

Figure S1. Top hit distribution of different plant species against *W. somnifera* TFs repertoire in non-redundant database on the basis of Blast homology.

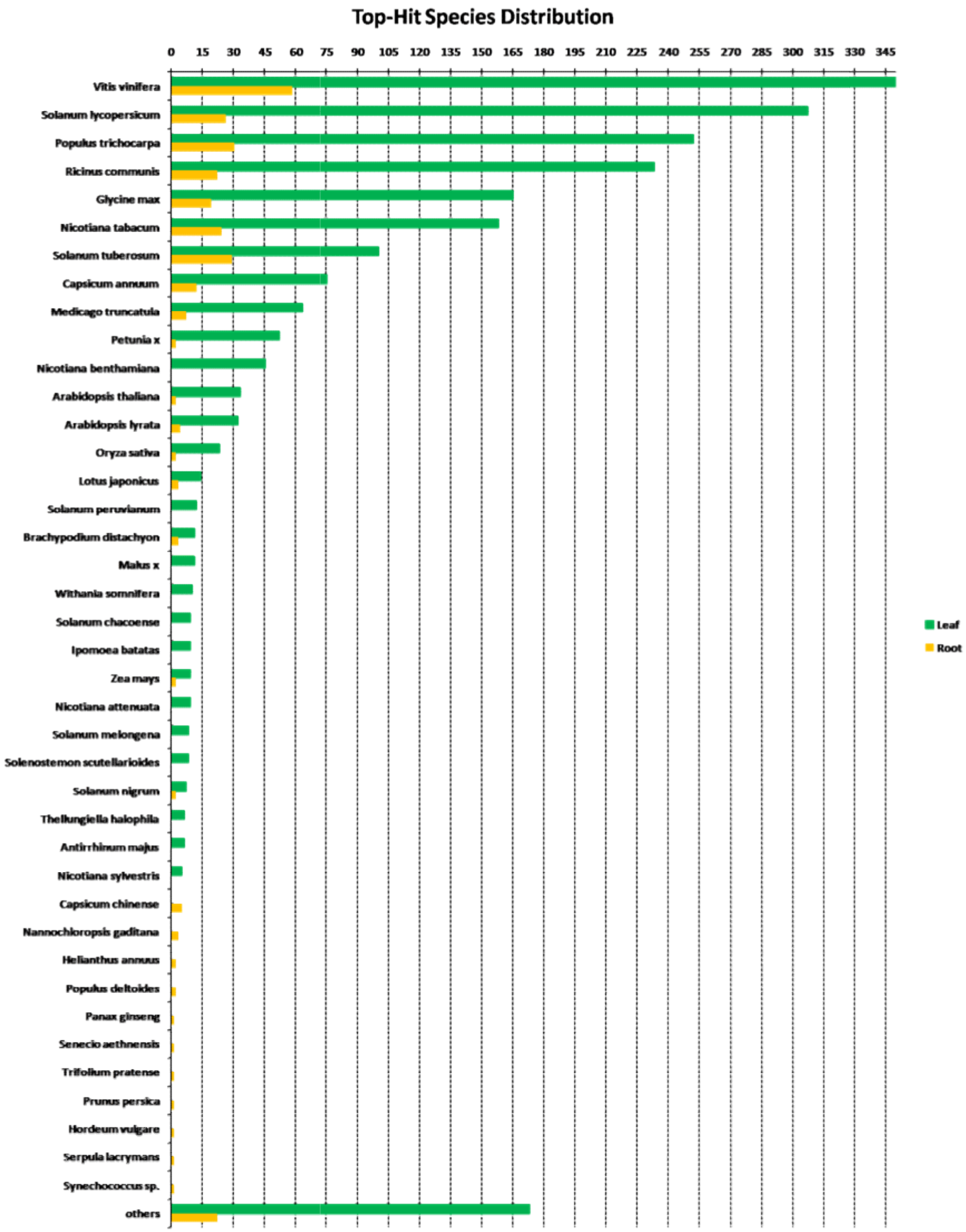

Table S1: Primers used in the study

Primer Name	Primer Sequence
WST7FLF	AAGCTTATGACGACAGGCATGGACTGC
WST7FLR	CTCGAGTTAAATAAGCTGCATTGGCAG
WST7RTF	GACTACTTGCACTGCATTGT
WST7RTR	CTCCTTCAACTTCTTATCCTTC
WST9FLF	GGATCCATGGAAGAGGACTCAACCTCG
WST9FLR	GAATTCTCATGTTTTCAGCTTCTTTTGC
WST9RTR	TCACCAGGAAGTAGCTTTCACCGT

Table S2: Overall description of transcripts involved in various biological processes with transcript count in *W. somnifera* leaf and root.

Process involved	No. of Leaf Transcripts	No. of Root Transcripts
Abscission	6	4
Aging	1	1
Anatomical structure development	309	262
Anatomical structure maturation	1	1
Anatomical structure morphogenesis	191	163
Aromatic compound biosynthetic process	1296	1082
Behavior	-	1
Biological regulation	428	330
Biosynthetic process	1484	1227
Carbohydrate metabolic process	167	86
Carboxylic acid metabolic process	100	71
Catabolic process	323	219
Cell communication	350	265
Cell cycle	161	124
Cell death	47	31
Cell differentiation	140	118
Cell growth	48	35
Cell-cell signaling	25	21
Cellular amino acid metabolic process	100	71
Cellular aromatic compound metabolic process	1616	1325
Cellular biosynthetic process	1329	1106
Cellular catabolic process	1	-
Cellular component assembly	7	-
Cellular component biogenesis	7	-
Cellular component organization or biogenesis	503	374
Cellular developmental process	140	118
Cellular homeostasis	15	11
Cellular macromolecular complex assembly	7	-
Cellular macromolecule biosynthetic process	1329	1106
Cellular macromolecule catabolic process	1	-
Cellular macromolecule metabolic process	1591	1304
Cellular metabolic process	1678	1367
Cellular nitrogen compound biosynthetic process	1296	1082
Cellular nitrogen compound metabolic process	1616	1325
Cellular process	1861	1506
Cellular protein catabolic process	1	-
Cellular protein metabolic process	337	246
Cellular protein modification process	266	195
Cellular response to stimulus	301	231
Cellular response to stress	1	-
Chromatin assembly	7	-
Chromatin assembly or disassembly	7	-
Chromatin organization	7	-
Chromosome organization	7	-

Death	47	31
Developmental maturation	1	1
Developmental process	510	438
Developmental process involved in reproduction	155	135
DNA conformation change	7	-
DNA metabolic process	356	268
DNA packaging	7	-
DNA repair	1	-
DNA replication	1	-
Embryo development	95	88
Establishment of localization	203	137
Flower development	154	134
Fruit development	1	1
Fruit ripening	1	1
Gene expression	1365	1127
Generation of precursor metabolites and energy	47	29
Growth	81	79
Heterocycle biosynthetic process	1296	1082
Heterocycle metabolic process	1616	1325
Homeostatic process	15	11
Lipid metabolic process	118	63
Localization	203	137
Macromolecular complex assembly	7	-
Macromolecular complex subunit organization	7	-
Macromolecule biosynthetic process	1329	1106
Macromolecule catabolic process	1	-
Macromolecule metabolic process	1640	1345
Macromolecule modification	266	195
Metabolic process	1786	1438
Modification-dependent macromolecule catabolic process	1	-
Modification-dependent protein catabolic process	1	-
Multicellular organismal development	507	432
Multicellular organismal process	541	450
Multi-multicellular organism process	12	7
Multi-organism process	12	7
Multi-organism reproductive process	12	7
Nitrogen compound metabolic process	1621	1329
Nucleic acid metabolic process	1504	1251
Nucleobase-containing compound biosynthetic process	1296	1082
Nucleobase-containing compound metabolic process	1616	1325
Nucleosome assembly	7	-
Nucleosome organization	7	-
Nucleotide-excision repair	1	-
Organelle organization	7	-
Organic acid metabolic process	100	71
Organic cyclic compound biosynthetic process	1296	1082
Organic cyclic compound metabolic process	1616	1325
Organic substance biosynthetic process	1329	1106

**Organic substance catabolic process**	1	-
**Organic substance metabolic process**	1740	1413
**Organonitrogen compound metabolic process**	100	71
**Oxoacid metabolic process**	100	71
**Photosynthesis**	55	14
**Pollination**	12	7
**Post-embryonic development**	352	310
**Primary metabolic process**	1737	1410
**Proteasomal protein catabolic process**	1	-
**Proteasomal ubiquitin-dependent protein catabolic process**	1	-
**Protein catabolic process**	1	-
**Protein complex assembly**	7	-
**Protein complex biogenesis**	7	-
**Protein complex subunit organization**	7	-
**Protein metabolic process**	440	328
**Protein modification process**	266	195
**Protein-DNA complex assembly**	7	-
**Protein-DNA complex subunit organization**	7	-
**Proteolysis**	1	-
**Proteolysis involved in cellular protein catabolic process**	1	-
**Regulation of biological process**	422	321
**Regulation of biological quality**	15	11
**Regulation of cellular process**	300	231
**Regulation of gene expression**	138	103
**Regulation of gene expression, epigenetic**	138	103
**Regulation of macromolecule metabolic process**	138	103
**Regulation of metabolic process**	138	103
**Reproduction**	323	280
**Reproductive process**	161	141
**Reproductive structure development**	155	135
**Reproductive system development**	155	135
**Response to abiotic stimulus**	532	376
**Response to biotic stimulus**	231	182
**Response to DNA damage stimulus**	1	-
**Response to endogenous stimulus**	329	241
**Response to external stimulus**	181	118
**Response to extracellular stimulus**	31	25
**Response to stimulus**	985	728
**Response to stress**	673	517
**RNA biosynthetic process**	1296	1082
**RNA metabolic process**	1296	1082
**Secondary metabolic process**	133	69
**Shoot system development**	154	134
**Signal transduction**	300	231
**Signaling**	323	248
**Single organism signaling**	323	248
**Single-multicellular organism process**	540	449
**Single-organism cellular process**	574	456

**Single-organism developmental process**	213	185
**Single-organism metabolic process**	236	143
**Single-organism process**	798	647
**Small molecule metabolic process**	100	71
**System development**	155	135
**Transcription from RNA polymerase II promoter**	2	-
**Transcription, DNA-dependent**	1296	1082
**Translation**	80	64
**Transport**	203	137
**Tropism**	77	42
**Ubiquitin-dependent protein catabolic process**	1	-

Table S3: An overview of predicted functions for *WsWRKY* contigs and transcripts.

Wrky contigs and Transcripts	Predicted functions
**wrcontigr 15284**	Abiotic stress (Oxidative Phosphate limitation and hormonal signalling like auxin and cytokinin)
**wrcontigr 7**	Abiotic stress (Drought and Salt, Cold and heat, oxidative), biotic and wounding hormonal signalling
**wrcontigr 11853**	Abiotic stress Drought and Salt, biotic stress and hormonal signalling
**wrcontigr 5**	Abiotic stress (oxidative, Drought and Salt) , biotic / wounding, Developmental process (seed dorm), Hormonal signalling (auxin and cytokinin)
**wrcontigr 8**	Abiotic stress, Drought and Salt, biotic stress and hormonal signalling
**wrcontigr 4118**	Defense response (response to bacteria, fungus, chitin hormonal signalling) (JA, SA) Development related (negative regulation leaf senescence)
**wrcontigr 16**	Defense response (response to bacteria, fungus, chitin hormonal sign) (JA, SA) Development related (negative regulation leaf senescence)
**wrcontigr 3**	Abiotic stress (Sugar Sensing and Metabolism), Biotic Stress/Wounding
**wrcontigr 11**	Abiotic stress (Drought and Salt, Cold, positive regulator of plant thermotolerance), Biotic , hormonal signalling (ethylene-response signal transduction pathway)
**wrcontigr 10**	Abiotic stress (Drought, Oxidative), Hormonal signalling (auxin and cytokinin)
**wrcontigr 10660**	Auxin and Cytokinin, Oxidative Stress
**wrcontigr 19921**	Abiotic stress (Drought and Salt, Cold and Heat, Oxidative, biotic stress / wounding stress , hormonal signalling
**wrcontigr 53266**	Developmental process
**wrcontigr 57273**	Abiotic stress (Sugar Sensing and Metabolism) biotic stress wounding
**wrcontigr 33223**	Abiotic stress (response to osmotic stress, response to salt stress, response to water deprivation, Oxidative stress), hormonal signalling
**wrcontigr 9817**	Abiotic stress (Sugar Sensing and Metabolism) biotic stress wounding
**wrcontigr 53369**	Abiotic stress (Drought and Salt, Oxidative, Defense response, hormonal signalling (gibberellin metabolic process)
**wrcontigr 2**	Cold response, hormonal signalling, pollen development and germination
**wrcontigr 229942**	Oxidative stress , seed dormancy
**wrcontigr 19**	ethylene responsive signal transduction pathway, positive regulation of plant thermotolerance
**wrcontigr 12**	wounding response, regulation of Defense response, response to chitin, response to SA
**wrcontigr 42054**	drought stress, hormonal signalling
**wrcontigr 9166**	Oxidative stress and hormonal signalling
**wrcontigr 1**	Drought and Salt stress, cold stress, biotic stress, hormonal signalling (auxin and cytokinin), developmental process
**wrcontigr 37881**	response to chitin, calmodulin binding
**wrcontigr 1753**	response to chitin, calmodulin binding
**wrcontigr 17**	Defense response regulation, response to chitin, SA response, wounding response
**wrcontigr 18**	Oxidative stress, response to hydrogen peroxide, response to ozone, response to SA, seed dormancy
**wrcontigr 4**	Drought and Salt, Cold stress, Oxidative, positive regulation of plant thermotolerance, biotic stress, ethylene -responsive signal transduction pathway
**wrcontigr 10778**	Oxidative stress, seed dormancy, response to hydrogen peroxide, response to ozone, response to SA
**wrcontigr 14**	calmodulin binding
**wrcontigr 14523**	Unknown
**wrcontigl 21995**	Abiotic stress (Sugar sensing and metabolism), embryo and pollen related development and metabolism and biosynthesis (glucuronoxylan and xylan biosynthesis)
**wrcontigl 14**	Abiotic stress (Oxidative) hormonal signalling

**wrcontigl 3628**	Abiotic stress (Oxidative, Drought and Salt , cold and heat) hormonal signalling, Developmental process seed dormancy, biotic/wounding stress
**wrcontigl 4**	Abiotic stress (Drought, heat, Oxidative), hormonal signalling and acts in calcium binding to mediate metabolic process
**wrcontigl 1**	Wounding response
**wrcontigl 7**	Abiotic stress (Drought and Salt, Cold, positive regulator of plant thermotolerance) Oxidative , Biotic , hormonal signalling (ethylene-response signal transduction pathway)
**wrcontigl 22188**	Abiotic stress (Drought and Salt, Oxidative), Hormonal signalling, biotic stress/ wounding
**wrcontigl 10**	Abiotic (Drought and Salt, Cold and Heat, Oxidative, biotic stress n wounding, hormonal signalling, Biotic stress (bacterial ,Defense response to fungus, regulation of Defense response), Hormonal signalling (SA)
**wrcontigl 8**	Abiotic stress (Drought and Salt, Cold, positive regulator of plant thermotolerance Oxidative) , Biotic , hormonal signalling (ethylene-responsive signal transduction pathway)
**wrcontigl 8810**	Abiotic stress (Drought and Salt), biotic stress, hormonal signalling
**wrcontigl 12**	Abiotic stress (Oxidative, Cold, heat stress, seed dormancy, hormonal signalling
**wrcontigl 6**	Developmental process, hormonal signalling
**wrcontigl 5**	response to chitin, calmodulin binding
**wrcontigl 28429**	Drought and salt stress, Defense response to bacteria, response to chitin and calmodulin binding
**wrcontigl 2**	Oxidative stress, phosphate limitation, hormonal signalling (auxin and cytokinin)
**wrcontigl 32030**	Unknown
**wrcontigl 43982**	Oxidative stress, developmental process, hormonal signalling
**wrcontigl 15084**	Plant defense, negative regulation of defense response to bacteria, regulation of defense response to fungi, response to ethylene, response to JA, response to SA, Biotic Stress/Wounding
**wrcontigl 9**	regulation of Defense response, response to chitin, response to wounding, response to SA,JA regulation of hydrogen peroxide metabolic process, response to fungus, auxin and cytokinin, pollen development, longitudinal axis specification, cell polarity, dev protein pollen development
**wrcontigl 2466**	Oxidative stress and developmental process
**wrcontigl 3**	Oxidative stress, seed dormancy (developmental process)
**wrcontigl 57368**	Regulation of defense response, response to chitin, response to SA, response to wounding
**wrcontigl 53584**	regulation of defense response, negative regulation of defense response to bacteria, regulation of defense response to fungus, response to ethylene, response to JA, response to SA, biotic stress and wounding
**wrcontigl 11**	unknown

Table S4: An overview of predicted functions for *WsWDR* contigs and transcripts.

Wdr contigs and Transcripts	Predicted functions
**wdcontigr 39817**	molecular scaffolds, U3 small nucleolar RNA-associated
**wdcontigr 19**	Wnt signaling pathway
**wdcontigr 9872**	Ribosome biogenesis in eukaryotes
**wdcontigr 7583**	sister chromatid cohesion, mRNA export from nucleus, protein import into nucleus, protein ubiquitination, regulation of cell cycle process, RNA methylation, rRNA processing
**wdcontigr 34745**	Cell cycle, Cell division, Mitosis
**wdcontigr 22846**	unknown
**wdcontigr 27**	May be involved in vesicle recycling
**wdcontigr 33962**	NET1-associated nuclear protein 1
**wdcontigr 40232**	ribosome biogenesis protein
**wdcontigr 14**	jasmonic acid (jasmonate, JA) signaling pathway, development related
**wdcontigr 12**	Ribosome biogenesis in eukaryotes
**wdcontigr 16569**	jasmonic acid (jasmonate, JA) signaling pathway, development related
**wdcontigr 4**	Autophagy
**wdcontigr 18**	Cell cycle, Cell division, Mitosis
**wdcontigr 36254**	myeloid differentiation and ribosome biogenesis
**wdcontigr 17**	Ubl conjugation pathway
**wdcontigr 13**	uncharacterized
**wdcontigr 32**	sister chromatid cohesion, mRNA export from nucleus, protein import into nucleus, protein ubiquitination, regulation of cell cycle process, RNA methylation, rRNA processing
**wdcontigr 7734**	Ribosome biogenesis in eukaryotes
**wdcontigr 20803**	growth and development through its role in cytokinesis and polarized cell expansion
**wdcontigr 55006**	unknown
**wdcontigr 43454**	Ribosome biogenesis, rRNA processing, Sensory transduction, Vision
**wdcontigr 25**	histone methylation
**wdcontigr 25**	sister chromatid cohesion, mRNA export from nucleus, protein import into nucleus, protein ubiquitination, regulation of cell cycle process, RNA methylation, rRNA processing
**wdcontigr 11858**	unknown
**wdcontigr 8**	sister chromatid cohesion, mRNA export from nucleus, protein import into nucleus, protein ubiquitination, regulation of cell cycle process, RNA methylation, rRNA processing
**wdcontigr 33452**	insulin signaling pathways
**wdcontigr 30**	uncharacterized
**wdcontigr 1576**	histone H3 lysine-4 trimethylation to control floral transition and plant development
**wdcontigr 38989**	May be involved in vesicle recycling
**wdcontigr 6567**	NET1-associated nuclear protein 1
**wdcontigr 26144**	histone methylation
**wdcontigr 49574**	unknown
**wdcontigr 7375**	Histone modification proteins
**wdcontigr 27154**	chromatin modification, histone acetylation, ubiquitin binding
**wdcontigr 26**	uncharacterized
**wdcontigr 31**	jasmonic acid (jasmonate, JA) signaling pathway, development related
**wdcontigr 56674**	Cell cycle, Cell division, Mitosis
**wdcontigr 9767**	Ribosome biogenesis in eukaryotes
**wdcontigr 12372**	Ubi conjugation pathway
**wdcontigr 53609**	regulate the activity of the USP12-UAF1 deubiquitinating enzyme complex

**wdcontigr 28**	uncharacterized
**wdcontigr 16668**	uncharacterized
**wdcontigr 16**	Ubi conjugation pathway
**wdcontigr 17493**	cold-responsive gene , metal ion binding, plant development
**wdcontigr 19574**	sister chromatid cohesion, mRNA export from nucleus, protein import into nucleus, protein ubiquitination, regulation of cell cycle process, RNA methylation, rRNA processing
**wdcontigr 33768**	fruit ripening
**wdcontigr 20**	anthocyanin and stress related
**wdcontigr 23**	Chromatin regulator
**wdcontigr 55226**	Fanconi anemia pathway
**wdcontigr 40572**	Abscisic acid signaling pathway, mRNA processing, mRNA splicing, mRNA transport, Transport, Ubi conjugation pathway
**wdcontigr 29**	abscisic acid-activated signaling pathway
**wdcontigr 16387**	Unknown
**wdcontigr 22**	Ubiquitin mediated proteolysis
**wdcontigr 10**	autophagy-related protein
**wdcontigr 41577**	Fanconi anemia pathway
**wdcontigr 55700**	Ribosome biogenesis in eukaryotes
**wdcontigr 1**	anthocyanin-containing compound biosynthetic process
**wdcontigr 11**	cold-responsive gene , metal ion binding, plant development
**wdcontigr 21**	basic wd-40 repeat
**wdcontigr 15**	Uncharacterized
**wdcontigr 2**	molecular scaffolds, U3 small nucleolar RNA-associated
**wdcontigr 23118**	Ubi conjugation pathway
**wdcontigr 47025**	Ubi conjugation pathway
**wdcontigr 5**	Unknown
**wdcontigr 16590**	Sister chromatid cohesion, mRNA export from nucleus, protein import into nucleus, protein ubiquitination, regulation of cell cycle process, RNA methylation, rRNA processing
**wdcontigr 25141**	Apoptosis, Autophagy, Cell cycle, Differentiation, DNA damage, Spermatogenesis
**wdcontigl 20**	Sister chromatid cohesion, mRNA export from nucleus, protein import into nucleus, protein ubiquitination, regulation of cell cycle process, RNA methylation, rRNA processing
**wdcontigl 1**	Uncharacterized
**wdcontigl 34292**	Sister chromatid cohesion, mRNA export from nucleus, protein import into nucleus, protein ubiquitination, regulation of cell cycle process, RNA methylation, rRNA processing
**wdcontigl 22**	uncharacterized
**wdcontigl 21448**	chromatin modification
**wdcontigl 18**	jasmonic acid (jasmonate, JA) signaling pathway, development related
**wdcontigl 35103**	histone methylation
**wdcontigl 20145**	unknown
**wdcontigl 14**	Ubi conjugation pathway
**wdcontigl 22523**	Ubiquitin mediated proteolysis
**wdcontigl 30117**	autophagy-related protein
**wdcontigl 22265**	Sister chromatid cohesion, mRNA export from nucleus, protein import into nucleus, protein ubiquitination, regulation of cell cycle process, RNA methylation, rRNA processing
**wdcontigl 26421**	myeloid differentiation and ribosome biogenesis
**wdcontigl 16**	Ubi conjugation pathway
**wdcontigl 23**	Sister chromatid cohesion, mRNA export from nucleus, protein import into nucleus, protein ubiquitination, regulation of cell cycle process, RNA methylation, rRNA processing
**wdcontigl 16612**	Unknown

**wdcontigl 12064**	Wnt signaling pathway
**wdcontigl 12**	Ribosome biogenesis in eukaryotes
**wdcontigl 39454**	Sister chromatid cohesion, mRNA export from nucleus, protein import into nucleus, protein ubiquitination, regulation of cell cycle process, RNA methylation, rRNA processing
**wdcontigl 26**	Cell cycle, Cell division, Mitosis
**wdcontigl 50339**	uncharacterized
**wdcontigl 29**	uncharacterized
**wdcontigl 27735**	Ribosome biogenesis in eukaryotes
**wdcontigl 34532**	unknown
**wdcontigl 58104**	Ribosome biogenesis in eukaryotes
**wdcontigl 938**	anthocyanin
**wdcontigl 30**	Regulate the activity of the USP12-UAF1 deubiquitinating enzyme complex
**wdcontigl 42964**	Sister chromatid cohesion, mRNA export from nucleus, protein import into nucleus, protein ubiquitination, regulation of cell cycle process, RNA methylation, rRNA processing
**wdcontigl 25**	Sister chromatid cohesion, mRNA export from nucleus, protein import into nucleus, protein ubiquitination, regulation of cell cycle process, RNA methylation, rRNA processing
**wdcontigl 58104**	Ribosome biogenesis in eukaryotes
**wdcontigl 6**	Fanconi anemia pathway
**wdcontigl 24670**	Abscisic acid signaling pathway, mRNA processing, mRNA splicing, mRNA transport, Transport, Ubl conjugation pathway
**wdcontigl 17**	anthocyanin-containing compound biosynthetic process
**wdcontigl 11732**	Cell cycle, Cell division, Mitosis
**wdcontigl 3**	Sister chromatid cohesion, mRNA export from nucleus, protein import into nucleus, protein ubiquitination, regulation of cell cycle process, RNA methylation, rRNA processing
